# Effects of a Hypnosis Session Before General Anesthesia on Postoperative Outcomes in Patients Who Underwent Minor Breast Cancer Surgery: The HYPNOSEIN Randomized Clinical Trial

**DOI:** 10.1001/jamanetworkopen.2018.1164

**Published:** 2018-08-17

**Authors:** Jibba Amraoui, Camille Pouliquen, Julien Fraisse, Jacques Dubourdieu, Sophie Rey Dit Guzer, Gilles Leclerc, Hélène de Forges, Marta Jarlier, Marian Gutowski, Jean-Pierre Bleuse, Chloé Janiszewski, Jésus Diaz, Philippe Cuvillon

**Affiliations:** 1 Department of Anesthesia, Montpellier Cancer Institute (ICM), University of Montpellier, Montpellier, France; 2 Department of Anesthesia, Paoli Calmette Institute, Marseille, France; 3 Biometrics Unit, Montpellier Cancer Institute (ICM), University of Montpellier, Montpellier, France; 4 Department of Anesthesia, Centre Hospitalier Universitaire Montpellier, University of Montpellier, Montpellier, France; 5 Department of Clinical Research, Montpellier Cancer Institute (ICM), University of Montpellier, Montpellier, France; 6 Department of Surgical Oncology, Montpellier Cancer Institute (ICM), University of Montpellier, Montpellier, France; 7 Department of Anesthesia, Centre Hospitalier Universitaire Nîmes, University of Montpellier, Nîmes, France

## Abstract

**Importance:**

Hypnosis is now widespread in medical practice and is emerging as an alternative technique for pain management and anxiety. However, its effects on postoperative outcomes remain unclear.

**Objective:**

To evaluate the efficacy of a preoperative hypnosis session for reducing postoperative breast pain in patients who underwent minor breast cancer surgery.

**Design, Setting, and Participants:**

The HYPNOSEIN prospective randomized clinical trial was conducted from October 7, 2014, to April 5, 2016. In this multicenter study in France, 150 women scheduled for minor breast cancer surgery were randomized between control and hypnosis arms, and 148 (71 control and 77 hypnosis) were included in the intent-to-treat analysis.

**Intervention:**

On the day of surgery, eligible patients were randomly assigned (1:1) to the control arm or the hypnosis arm. Patients (but not the care teams) were blinded to the arm to which they were assigned. A 15-minute hypnosis session before general anesthesia in the operating room was performed in the hypnosis arm.

**Main Outcomes and Measures:**

The primary end point was breast pain reduction (by 2 on a visual analog scale), assessed immediately before discharge from the postanesthesia care unit (PACU). Secondary end points were nausea/vomiting, fatigue, comfort/well-being, anxiety, and PACU length of stay, assessed at different times until postoperative day 30.

**Results:**

The median patient age was 57 years (range, 33-79 years) in the control arm and 53 years (range, 20-84 years) in the hypnosis arm. Baseline characteristics were similar in the 2 arms. The median duration of the hypnosis session was 6 minutes (range, 2-15 minutes). The use of intraoperative opioids and hypnotics was lower in the hypnosis arm. The mean (SD) breast pain score (range, 0-10) was 1.75 (1.59) in the control arm vs 2.63 (1.62) in the hypnosis arm (*P* = .004). At PACU discharge and with longer follow-up, no statistically significant difference in breast pain was reported. Fatigue was significantly lower in the hypnosis arm on the evening of surgery (mean [SD] score, 3.81 [2.15] in the control arm vs 2.99 [2.56] in the hypnosis arm; *P* = .03). The median PACU length of stay was 60 minutes (range, 20-290 minutes) in the control arm vs 46 minutes (range, 5-100 minutes) in the hypnosis arm (*P* = .002). Exploratory analyses according to patient perception of whether she received hypnosis showed significantly lower fatigue scores in the perceived hypnosis subgroup on the evening of surgery (mean [SD], 4.13 [2.26] for no perceived hypnosis vs 2.97 [2.42] for perceived hypnosis; *P* = .01). Anxiety was also significantly lower on the evening of surgery in the perceived hypnosis subgroup (mean [SD], 0.75 [1.64] for perceived hypnosis vs 1.67 [2.29] for no perceived hypnosis; *P* = .03).

**Conclusions and Relevance:**

The results of this study do not support a benefit of hypnosis on postoperative breast pain in women undergoing minor breast cancer surgery. However, other outcomes seem to be improved, which needs to be confirmed by further studies.

**Trial Registration:**

EudraCT Identifier: 2014-A00681-46 and ClinicalTrials.gov Identifier: NCT03253159

## Key Points

**Question:**

What is the benefit of a short hypnosis session before general anesthesia on postoperative outcomes (pain, nausea/vomiting, fatigue, comfort/well-being, anxiety, postanesthesia care unit length of stay, and patient satisfaction) in patients who underwent minor breast cancer surgery?

**Findings:**

In this randomized clinical trial, 150 women were randomized to receive hypnosis or a control group, and the mean breast pain score before discharge was 1.75 in the control arm vs 2.63 in the hypnosis arm. At discharge, no statistically significant difference in breast pain was reported.

**Meaning:**

No benefit of hypnosis was found on postoperative breast pain; however, hypnosis seems to have other benefits regarding fatigue, anxiety, and patient satisfaction.

## Teaser

This randomized clinical trial compares the efficacy of preoperative hypnosis vs usual care for reducing postoperative breast pain in patients undergoing breast cancer tumor removal with or without limited axillary node dissection.

## Introduction

Breast cancer is the most frequent cancer among women, with psychological, professional, and social effects.^1,2^ Diagnosis and treatment, as well the consequences on womanhood and everyday life, are often harsh. In this “hostile” environment, most women undergo breast surgery, which can lead to even more stress.^3^ Studies^4,5^ have reported that within the first postoperative days patients experience moderate or severe pain (10%-20%), nausea/vomiting (10%-15%), drowsiness (10%), sore throat (15%), and hoarseness, causing anxiety and discomfort. Therefore, reduction of these postoperative complications remains a medical challenge, with economic issues that include delayed discharge from the postanesthesia care unit (PACU) and center, medical and pharmacological rescue, and readmission.

Several strategies, such as premedication or pharmacological multimodal approaches, have been proposed to reduce these effects.^6,7^ Nonpharmacological alternatives, including music, relaxation therapy, or medical hypnosis, have been shown to decrease perioperative anxiety, pain, medication requirement, and nausea/vomiting.^8,9,10,11^ Among them, hypnosis seems to be a convenient method, with advantages that include an inexpensive and simple technique with no specific adverse effects. Observational studies and meta-analyses have reported the benefit of hypnosis on postoperative pain and other postoperative adverse effects, anesthetic intake, and duration of stay in the PACU.^12,13,14^ In 2007, Montgomery et al^12^ showed that a brief hypnosis session (15 minutes) performed within 1 hour before breast surgery reduced both adverse effects and cost. Hypnosis has become popular in numerous hospitals, leading to enhanced empathy and verbal and nonverbal communication in the preoperative and intraoperative settings.^10,12,13,14^ However, these hypnosis techniques and attitudes have not been validated by rigorous randomized studies to date.

The HYPNOSEIN prospective single-blind randomized clinical trial evaluated the effect of hypnosis performed immediately before general anesthesia on main adverse effects in patients scheduled for day-case breast cancer surgery. The primary objective of this multicenter study in France was to evaluate the efficacy of a preoperative hypnosis session for reducing postoperative breast pain in patients who underwent minor breast cancer surgery, assessed using a visual analog scale (VAS) in the PACU. We also investigated the effect of hypnosis on nausea/vomiting, fatigue, comfort/well-being, and anxiety.

## Methods

### Study Design and Setting

This multicenter, prospective, randomized, single-blind, phase 3 clinical trial was conducted in the following centers: Montpellier Cancer Institute (ICM) and Montpellier University Hospital, Montpellier, France, and Paoli-Calmettes Institute, Marseille, France. The study was approved by the local institutional and ethics committee (Comité de Protection des Personnes [CPP], Sud Méditerrannée III, Nîmes, France). It was conducted in accord with the ethical standards of the Declaration of Helsinki^15^ and the Good Clinical Practice requirements.^16^ All participants provided written informed consent before the study began. This study followed the Consolidated Standards of Reporting Trials (CONSORT) reporting guidelines. The complete trial protocol is available in the Supplement.

### Study Population

Patients were included in the study if they were women older than 18 years with an indication for minor breast cancer surgery (cancer tumorectomy or tumorectomy with limited axillary node dissection). They had to be scheduled for day-case breast surgery (ambulatory, discharge on the same day, or discharge on the following day), with general anesthesia. Patients were excluded if they had an American Society of Anesthesiologists score of 4 or higher, body mass index (calculated as weight in kilograms divided by height in meters squared) less than 15 or greater than 45, or an indication for major surgery (mastectomy, bilateral surgery, full axillary dissection, major breast reconstruction, lumpectomy, or previous duration of surgery exceeding 2 hours). Also excluded were patients who refused hypnosis or had undergone previous surgery with hypnosis and patients with psychiatric disorders, chronic pain, or the use of therapeutic opioids for more than 3 months.

### Randomization and Blinding

On the day of surgery, eligible patients were randomly assigned (1:1) to the control arm or the hypnosis arm. Patients (but not the care teams) were blinded to the arm to which they were assigned. To reduce bias (excessive empathy in the control arm), 2 different anesthesiologist teams were involved in patient care. Patients in the control arm were managed by caregivers without formal hypnosis training, and patients in the hypnosis arm were cared for by a dedicated team of staff trained in hypnosis.

### Intervention Preoperative Preparation and Hypnosis

No premedication was given, and music therapy was not allowed. Patients in the control arm were prepared for surgery by the control team, with no specific recommendation on wording or nonverbal communication, and standard general anesthesia was used. In the hypnosis arm, patients were prepared for surgery by the hypnosis team, who started verbal and nonverbal communication immediately. A short individual hypnosis session (≤15 minutes) that was personalized to each patient was performed in all centers by a trained anesthesiologist who had been practicing the technique for more than 1 year. It was recommended that the anesthesiologist use sensorial language and paraverbal and rewording techniques to promote patient comfort/well-being according to her choice of a safe place or leisure activity. Family concerns and negative topics were avoided. During the hypnosis session, only the anesthesiologist talked with the patient. When the physician thought that the patient was ready for anesthesia, pharmacological anesthesia induction was performed in a manner similar to that for the control arm.

### Anesthetic Management, Analgesia, and Postoperative Care

Preoperative preparation, analgesia, surgery, and postoperative care were similar in the 2 arms in accord with the standards of the participating centers. Anesthesia induction was started intravenously with lidocaine hydrochloride (1 mg/kg), propofol (2-3 mg/kg), sufentanil citrate (0.1-0.2 μg/kg), and *cis*-atracurium (0.3-0.5 mg/kg), if necessary. The airway was secured with an endotracheal or supraglottic tube, and the lungs were ventilated with a mix of oxygen and air (50%-50%). The tidal volume was set to 6 to 8 mL/kg of ideal body weight. Anesthesia was maintained using sevoflurane (1%-2%), and additional intravenous sufentanil citrate (5 μg) was administered during surgery if the heartbeat or systolic blood pressure increased more than 20%. During surgery, analgesics were administered, including acetaminophen (1 g), ketoprofen (50 mg), and ketamine hydrochloride (0.1-0.2 mg/kg). No local anesthetic solution was administered.

In the PACU, rescue analgesia (intravenous tramadol hydrochloride [50 mg]) was given to patients whose pain exceeded 3 on a 10-point numeric scale. If a score above 3 remained after 30 minutes, morphine sulfate titration (2 mg for body mass <60 kg and 3 mg for body mass ≥60 kg) at 5-minute intervals was administered until a score of 3 or lower was reached. In the first postoperative 48 hours, all patients received acetaminophen (1 g) and ketoprofen (50 mg) at 6-hour intervals by mouth.

As nausea/vomiting prophylaxis, all patients with an Apfel score exceeding 2 were given intravenous dexamethasone sodium phosphate (4 mg) and droperidol (1.25 mg) after anesthesia induction.^17,18^ To treat nausea/vomiting after extubation, patients received intravenous ondansetron hydrochloride (4 mg). When necessary, oral ondansetron was administered during the first 48 hours after surgery (4 mg every 6 hours).

### Clinical Assessment of Outcomes

The primary end point was breast pain reduction, assessed immediately before discharge from the PACU by 2 on the VAS. Secondary end points were evaluation on the VAS of the following: postsurgical nausea/vomiting, fatigue, comfort/well-being, anxiety, PACU length of stay, operative time, use and dose of antiemetics, analgesic consumption, and number of failed day-case surgical procedures. Outcomes were recorded using the VAS (range, 0-10) by an independent clinical research associate before surgery, immediately before PACU discharge, at patient discharge on the evening of surgery, and at days 1, 7, and 30 after surgery. Assessments of outcomes in the PACU were performed by a blinded nurse. Patient satisfaction with care was evaluated the day after surgery on a scale of 0 to 10.

### Statistical Analysis

Randomization was stratified according to center. The sample size calculation was based on a difference of at least 2 on the VAS in the PACU between the 2 arms in terms of pain severity. To detect such a difference with σ of 3.5, 2-sided α risk of 5%, and power of 90% (β = .10), 66 patients per arm were required. Considering 10% of nonevaluable patients, a recruitment total of 150 patients (75 per arm) was planned.^19^

All analyses were performed on an intent-to-treat basis according to the statistical analysis plan (Supplement). No imputation method was used in the case of missing data. Data were recorded by treatment group. Continuous variables were described using means (SDs) and medians (ranges). For categorical variables, frequencies and percentages were computed. For both quantitative and qualitative variables, missing data were reported. To compare the distribution of continuous variables, *t* test or Kruskal-Wallis test was used. Categorical variables were compared using χ^2^ test or Fisher exact test.

An exploratory subgroup analysis was conducted to assess the effect of treatment group perception on end points. During their PACU stay, patients were asked whether they thought that they had received hypnosis (“Do you think you received hypnosis before anesthesia?”), and 2 subgroups (perceived hypnosis and no perceived hypnosis) were considered based on their perception. Accordingly, the pain, fatigue, comfort/well-being, and anxiety in each subgroup were described.

All tests were 2-sided, and *P* < .05 was considered statistically significant. Statistical analyses were performed using a software program (Stata, version 13.0; StataCorp LP).

## Results

### Patients

Between October 7, 2014, and April 5, 2016, a total of 150 patients were randomized, and 73 and 77 were allocated to the control and hypnosis arms, respectively (Figure). Two patients were excluded from the safety and efficacy analysis (1 patient was not treated according to the protocol, and 1 patient did not meet an eligibility criterion). Patient characteristics were well balanced between the 2 arms (Table 1). Briefly, the mean patient age was 57 years (range, 33-79 years) in the control arm and 53 years (range, 20-84 years) in the hypnosis arm. Most patients had undergone previous surgery (91.5% [65 of 71] in the control arm and 83.1% [64 of 77] in the hypnosis arm). Pain, nausea/vomiting, fatigue, comfort/well-being, and anxiety assessed at baseline immediately before entering the operating room were similar for patients in the 2 arms.

**Figure.**
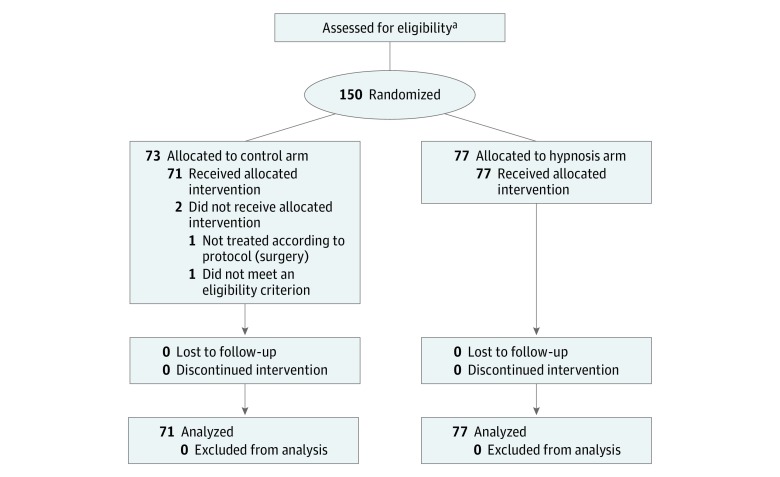
CONSORT Diagram of the Study CONSORT indicates Consolidated Standards of Reporting Trials. ^a^Patients screened were reported in 2 participating centers and not in the third (center 3).

**Table 1.** Patient Characteristics at Baseline^a^

Variable	Control Arm (n = 71)	Hypnosis Arm (n = 77)
Age, median (range), y	57 (33-79)	53 (20-84)
BMI, median (range)	23 (15-32)	23 (16-38)
Apfel score, median (range)	2 (1-4)	2 (1-4)
Missing	(n = 6)	(n = 7)
APAIS score, median (range)	14 (6-26)	13 (6-25)
Missing	(n = 3)	(n = 1)
ASA score, median (range)	1 (1-3)	1 (1-3)
Education, No./total No. (%)		
<Bachelor degree	18/68 (26.5)	23/72 (31.9)
Bachelor degree	17/68 (25.0)	12/72 (16.7)
Postgraduate	33/68 (48.5)	37/72 (51.4)
Missing	(n = 3)	(n = 5)
Occupation, No./total No. (%)		
Active	31/71 (43.7)	41/75 (54.7)
Inactive, unemployed, retired	40/71 (56.3)	34/75 (45.3)
Missing	(n = 0)	(n = 2)
Medical history, No. (%)	66 (93.0)	68 (88.3)
Previous surgery, No. (%)	65 (91.5)	64 (83.1)
Premedication, No./total No. (%)	18/67 (26.9)	11/74 (14.9)
Missing	(n = 4)	(n = 3)
VAS score, mean (SD)^b^		
Breast pain	0.39 (0.72)	0.51 (1.23)
General pain	1.44 (1.78)	1.45 (2.05)
Nausea/vomiting	0.24 (0.98)	0.22 (0.80)
Fatigue	2.11 (2.05)	2.47 (2.33)
Comfort/well-being	7.65 (2.04)	7.42 (2.13)
Anxiety	4.48 (2.73)	3.84 (2.70)

Abbreviations: APAIS, Amsterdam Preoperative Anxiety and Information Scale; ASA, American Society of Anesthesiologists; BMI, body mass index (calculated as weight in kilograms divided by height in meters squared); VAS, visual analog scale.

^a^ All baseline characteristics were well balanced between the 2 arms.

^b^ Measured immediately before entering the operating room using a VAS graded from 0 to 10.

### Hypnosis Session

In the hypnosis arm, the median duration of the hypnosis session was 6 minutes (range, 2-15 minutes). The use of intraoperative opioids and hypnotics was lower in the hypnosis arm. After surgery, 25.0% (14 of 56) of patients in the control arm thought that they had received hypnosis, and 21.7% (15 of 69) of patients in the hypnosis arm thought that they had not received hypnosis.

### Treatments

Almost all patients (100% [71 of 71] in the control arm and 98.7% [76 of 77] in the hypnosis arm) underwent tumorectomy or quadrantectomy (Table 2). The median operative time was the same in the control arm (50 minutes [range, 15-193 minutes]) and the hypnosis arm (50 minutes [range, 15-220 minutes]). Drug consumption was similar overall except for doses of propofol and sufentanil, which were both lower in the hypnosis arm: the doses in the control vs hypnosis arms were 240 mg (range, 120-450 mg) vs 200 mg (range, 100-450 mg) (*P* = .01) for propofol and 19 μg (range, 10-30 μg) vs 15 μg (range, 10-25 μg) (*P* = .05) for sufentanil citrate. The proportion of patients who received lidocaine was lower in the control arm than in the hypnosis arm (46.5% [33 of 71] vs 68.8% [53 of 77], *P* = .008). The choice of airway management technique was also significantly different, favoring a noninvasive laryngeal mask in the hypnosis arm (70.1% [54 of 77]) vs in the control arm (53.5% [38 of 71]) (*P* = .04). The median PACU length of stay was 60 minutes (range, 20-290 minutes) in the control arm vs 46 minutes (range, 5-100 minutes) in the hypnosis arm (*P* = .002).

**Table 2.** Surgical, Anesthesia, and Postoperative Data

Variable	Control Arm (n = 71)	Hypnosis Arm (n = 77)	*P* Value
**Surgical Data**			
Surgery type, No. (%)			
Tumorectomy or quadrantectomy	71 (100)	76 (98.7)	>.99
Oncoplasty	0	1 (1.3)	
Operative time, median (range), min	50 (15-193)	50 (15-220)	.77
Missing	(n = 8)	(n = 6)	
**Anesthesia Data**			
Anesthesia duration, median (range), min	95 (25-232)	87 (41-210)	.27
Missing	(n = 15)	(n = 9)	
Intubation type, No. (%)			
Orotracheal	33 (46.5)	23 (29.9)	.04
Laryngeal mask	38 (53.5)	54 (70.1)	
Midazolam hydrochloride, No. (%)	4 (5.6)	6 (7.8)	.75
Median (range), mg	1 (1-2)	1 (1-2)	.83
Propofol, No. (%)	71 (100)	77 (100)	>.99
Median (range), mg	240 (120-450)	200 (100-450)	.01
Missing	(n = 2)	(n = 0)	
Sufentanil citrate, No. (%)	60 (84.5)	64 (83.1)	>.99
Median (range), μg	19 (10-30)	15 (10-25)	.05
Morphine sulfate reinjection, No. (%)	22 (31.0)	16 (20.8)	.19
Median (range), μg	7.5 (0.1-460.0)	5.0 (0.1-15.0)	.11
Ketamine hydrochloride, No. (%)	37 (52.1)	49 (63.6)	.18
Median (range), mg	15 (10-30)	15 (10-40)	.91
Lidocaine hydrochloride, No. (%)	33 (46.5)	53 (68.8)	.008
Median (range), mg	50 (10-80)	50 (30-150)	.18
Curare, No. (%)	12 (16.9)	9 (11.7)	.48
Median (range), mg	6 (5-10)	6 (4-20)	.90
Missing	(n = 0)	(n = 1)	
PONV prophylaxis, No. (%)	34 (47.9)	19 (24.7)	.004
**Postoperative Data**			
PACU length of stay, median (range), min	60 (20-290)	46 (5-100)	.002
Missing	(n = 4)	(n = 3)	
Antiemetic treatment, No./total No. (%)	4/69 (5.8)	8/77 (10.4)	.38
Missing	(n = 2)	(n = 0)	
Including ondansetron hydrochloride, No./total No. (%)	3/4 (75.0)	7/8 (87.5)	>.99
Analgesic consumption, No./total No. (%)	32/69 (46.4)	34/77 (44.2)	.87
Missing	(n = 2)	(n = 0)	
Including morphine sulfate, No./total No. (%)	2/32 (6.3)	6/33 (18.2)	.26
Missing	(n = 0)	(n = 1)	
Patient discharge, No./total No. (%)			
Day 0	52/70 (74.3)	61/77 (79.2)	.56
Day 1^a^	13/70 (18.6)	12/77 (15.6)	.91
Missing	(n = 2)	(n = 0)	
Patient satisfaction regarding anesthesia care and management, mean (SD)^b^	8.9 (1.5)	9.5 (1.1)	.02
Missing	(n = 7)	(n = 2)	

Abbreviations: PACU, postanesthesia care unit; PONV, postoperative nausea/vomiting.

^a^ Patients discharged on day 1 were not discharged on day 0 only for logistic or familial reasons. No complications were reported.

^b^ Patient satisfaction was evaluated on the day after surgery.

The postoperative nausea/vomiting (PONV) prophylaxis was different in the 2 arms: 47.9% (34 of 71) of patients received prophylaxis in the control arm vs 24.7% (19 of 77) in the hypnosis arm (*P* = .004), which prohibited interpretation of these results and analysis of the effect of hypnosis on PONV. One month after surgery, no serious adverse event or medical or surgical complication had been reported in either of the 2 arms.

### Postoperative Pain Primary End Point

The mean (SD) breast pain score (range, 0-10), assessed immediately before PACU discharge, was 1.75 (1.59) in the control arm vs 2.63 (1.62) in the hypnosis arm (*P* = .004), favoring the control arm (difference, −0.88; 95% CI, −1.45 to −0.29) (Table 3). At discharge on the evening of surgery and with longer follow-up (postoperative days 1, 7, and 30), no difference in the breast and general pain scores was observed between the 2 arms.

**Table 3.** Patient Outcomes Assessed on the Day of Surgery (in the PACU and at Patient Discharge on the Evening of Surgery) and on Day 1 in the Control and Hypnosis Arms^a^

Variable	Control Arm (n = 71)		Hypnosis Arm (n = 77)		*P* Value	Difference (95% CI)^b^
	Median (Range)	Mean (SD)	Median (Range)	Mean (SD)		
**Breast Pain**						
PACU	2 (0 to 6)	1.75 (1.59)	3 (0 to 7)	2.63 (1.62)	.004	−0.88 (−1.45 to −0.29)
Missing	(n = 18)		(n = 7)			
Evening of surgery	2 (0 to 6)	2.14 (1.68)	2 (0 to 6)	2.27 (1.61)	.62	−0.13 (−0.67 to 0.41)
Missing	(n = 2)		(n = 2)			
Day 1	1 (0 to 8)	1.45 (1.58)	2 (0 to 7)	1.84 (1.77)	.21	−0.39 (−0.95 to 0.27)
Missing	(n = 5)		(n = 1)			
**General Pain**						
PACU	0 (0 to 4)	0.75 (1.19)	0 (0 to 6)	1.00 (1.57)	.77	−0.25 (−0.76 to 0.27)
Missing	(n = 18)		(n = 7)			
Evening of surgery	0 (0 to 6)	0.88 (1.57)	0 (0 to 7)	0.88 (1.45)	.87	0.00 (−0.49 to 0.50)
Missing	(n = 2)		(n = 2)			
Day 1	0 (0 to 5)	0.64 (1.13)	0 (0 to 7)	0.87 (1.72)	.88	−0.24 (−0.72 to 0.26)
Missing	(n = 5)		(n = 1)			
**Fatigue**						
PACU	4 (0 to 10)	3.81 (2.56)	4 (0 to 10)	3.66 (2.40)	.76	0.15 (−0.74 to 1.04)
Missing	(n = 18)		(n = 6)			
Evening of surgery	4 (0 to 9)	3.81 (2.15)	3 (0 to 9)	2.99 (2.56)	.03	0.82 (0.03 to 1.61)
Missing	(n = 3)		(n = 3)			
Day 1	3 (0 to 7)	2.79 (2.03)	2 (0 to 9)	2.45 (2.37)	.24	0.34 (−0.40 to 1.08)
Missing	(n = 5)		(n = 1)			
**Comfort/Well-being**						
PACU	8 (0 to 10)	7.43 (2.26)	8 (0 to 10)	7.15 (2.36)	.57	0.28 (−0.55 to 1.11)
Missing	(n = 18)		(n = 6)			
Evening of surgery	7 (0 to 10)	6.91 (2.26)	8 (1 to 10)	7.31 (2.16	.31	−0.40 (−1.12 to 0.33)
Missing	(n = 2)		(n = 2)			
Day 1	8 (0 to 10)	7.18 (2.53)	8 (1 to 10)	7.71 (1.96)	.39	−0.53 (−1.27 to 0.22)
Missing	(n = 5)		(n = 1)			
**Anxiety**						
PACU	0 (0 to 9)	1.68 (2.23)	0 (0 to 7)	1.14 (1.78)	.35	0.54 (−0.18 to 1.25)
Missing	(n = 18)		(n = 6)			
Evening of surgery	0 (0 to 9)	1.41 (2.07)	0 (0 to 10)	0.91 (1.85)	.11	0.50 (−0.15 to 1.14)
Missing	(n = 2)		(n = 2)			
Day 1	0 (0 to 10)	1.17 (2.13)	0 (0 to 9)	1.08 (2.14)	.56	0.09 (−0.62 to 0.81)
Missing	(n = 5)		(n = 1)			

Abbreviation: PACU, postanesthesia care unit.

^a^ All variables were assessed using a visual analog scale scored from 0 to 10 (0 indicates not at all, and 10 indicates unbearable pain, extreme nausea/vomiting, extreme fatigue requiring bed rest, and maximal possible anxiety). For comfort/well-being, 0 indicates very uncomfortable, and 10 indicates maximal comfort desired.

^b^ Difference is the visual analog scale score in the control arm minus the visual analog scale score in the hypnosis arm.

### Secondary End Points

The mean (SD) VAS scores for fatigue in the PACU were 3.81 (2.56) in the control arm vs 3.66 (2.40) in the hypnosis arm, which was not significantly different (difference, 0.15; 95% CI, −0.74 to 1.04) (Table 3). The mean (SD) VAS score for fatigue on the evening of surgery was significantly lower in the hypnosis arm (3.81 [2.15] in the control arm vs 2.99 [2.56] in the hypnosis arm) (difference, 0.82, 95% CI, 0.03-1.61; *P* = .03). Levels of comfort/well-being and anxiety immediately after surgery were similar in the 2 arms. At days 1, 7, and 30 after surgery, there was a tendency toward lower levels of fatigue, comfort/well-being, and anxiety in the hypnosis arm compared with the control arm, but the differences were not statistically significant. The mean (SD) perioperative care patient satisfaction evaluated on the day after surgery was 8.9 (1.5) in the control arm vs 9.5 (1.1) in the hypnosis arm (*P* = .02) (Table 2).

Exploratory analyses were conducted according to patient perception of whether she received hypnosis. Patient characteristics were well balanced for the 2 subgroups of perceived hypnosis (68 of 125 patients [54.4%]) and no perceived hypnosis (57 of 125 patients [45.6%]). Significantly lower fatigue scores were reported in the perceived hypnosis subgroup on the evening of surgery (mean [SD], 4.13 [2.26] for no perceived hypnosis vs 2.97 [2.42] for perceived hypnosis) (difference, 1.16; 95% CI, 0.31-2.00; *P* = .01) and on day 1 after surgery (mean [SD], 3.09 [2.15] for no perceived hypnosis vs 2.33 [2.37] for perceived hypnosis; difference, 0.76; 95% CI, −0.06 to 1.58; *P* = .048) (Table 4). The level of anxiety was also significantly lower on the evening of surgery in the perceived hypnosis subgroup (mean [SD], 1.67 [2.29] for no perceived hypnosis vs 0.75 [1.64] for perceived hypnosis (difference, 0.92; 95% CI, 0.22-1.62; *P* = .03). At days 1, 7, and 30 after surgery, levels of fatigue, comfort/well-being, and anxiety were not statistically different in the 2 treatment perception subgroups.

**Table 4.** Patient Outcomes Assessed on the Day of Surgery (in the PACU and at Patient Discharge on the Evening of Surgery) and on Day 1 in the Control and Hypnosis Arms, Stratified by Treatment Group Perception^a^

Variable	Treatment Group Perception				*P* Value	Difference (95% CI)^b^
	No Perceived Hypnosis Subgroup (n = 57)		Perceived Hypnosis Subgroup (n = 68)			
	Median (Range)	Mean (SD)	Median (Range)	Mean (SD)		
**Breast Pain**						
PACU	2 (0 to 5)	1.80 (1.64)	3 (0 to 7)	2.78 (1.67)	.005	−0.98 (−1.61 to −0.34)
Missing	(n = 7)		(n = 10)			
Evening of surgery	2 (0 to 6)	2.07 (1.73)	2 (0 to 6)	2.52 (1.54)	.10	−0.45 (−1.03 to 0.13)
Missing	(n = 0)		(n = 0)			
Day 1	2 (0 to 8)	1.75 (1.62)	1 (0 to 7)	1.67 (1.70)	.65	0.08 (−0.52 to 0.68)
Missing	(n = 2)		(n = 2)			
**General Pain**						
PACU	0 (0 to 4)	0.71 (1.20)	0 (0 to 6)	1.09 (1.66)	.48	−0.38 (−0.94 to 0.18)
Missing	(n = 6)		(n = 11)			
Evening of surgery	0 (0 to 6)	0.58 (1.07)	0 (0 to 7)	1.15 (1.70)	.19	−0.57 (−1.08 to −0.05)
Missing	(n = 0)		(n = 0)			
Day 1	0 (0 to 7)	0.78 (1.47)	0 (0 to 6)	0.74 (1.41)	.88	0.04 (−0.48 to 0.56)
Missing	(n = 2)		(n = 2)			
**Fatigue**						
PACU	4 (0 to 10)	4.20 (2.59)	3 (0 to 10)	3.26 (2.26)	.07	0.94 (0.02 to 1.86)
Missing	(n = 6)		(n = 10)			
Evening of surgery	4 (0 to 9)	4.13 (2.26)	3 (0 to 8)	2.97 (2.42)	.01	1.16 (0.31 to 2.00)
Missing	(n = 1)		(n = 1)			
Day 1	3 (0 to 8)	3.09 (2.15)	2 (0 to 9)	2.33 (2.37)	.048	0.76 (−0.06 to 1.58)
Missing	(n = 2)		(n = 2)			
**Comfort/Well-being**						
PACU	8 (0 to 10)	7.35 (2.08)	7 (0 to 10)	7.10 (2.48)	.77	0.25 (−0.63 to 1.13)
Missing	(n = 6)		(n = 10)			
Evening of surgery	7 (0 to 10)	6.63 (2.01)	8 (1 to 10)	7.13 (2.30)	.14	−0.50 (−1.27 to 0.27)
Missing	(n = 0)		(n = 0)			
Day 1	7 (0 to 10)	7.00 (2.20)	8 (1 to 10)	7.68 (2.18)	.05	−0.68 (−1.47 to 0.11)
Missing	2		2			
**Anxiety**						
PACU	0 (0 to 9)	1.69 (2.34)	0 (0 to 5)	1.05 (1.58)	.37	0.64 (−0.12 to 1.38)
Missing	(n = 6)		(n = 10)			
Evening of surgery	0 (0 to 9)	1.67 (2.29)	0 (0 to 10)	0.75 (1.64)	.03	0.92 (0.22 to 1.62)
Missing	(n = 0)		(n = 0)			
Day 1	0 (0 to 10)	1.53 (2.42)	0 (0 to 8)	0.82 (1.79)	.16	0.71 (−0.05 to 1.47)
Missing	(n = 2)		(n = 2)			

Abbreviation: PACU, postanesthesia care unit.

^a^ All variables were assessed using a visual analog scale scored from 0 to 10 (0 indicates not at all, and 10 indicates unbearable pain, extreme nausea/vomiting, extreme fatigue requiring bed rest, and maximal possible anxiety). For comfort/well-being, 0 indicates very uncomfortable, and 10 indicates maximal comfort desired.

^b^ Difference is the visual analog scale score in the no perceived hypnosis subgroup minus the visual analog scale score in the perceived hypnosis subgroup.

## Discussion

We report the results of a multicenter, prospective, randomized, single-blind phase 3 clinical trial evaluating the benefit of preoperative hypnosis sessions in day-case minor breast cancer surgery. There was no benefit of a hypnosis session performed immediately before general anesthesia induction on postoperative breast pain in these patients. However, patients who thought that they had received hypnosis had significantly lower postoperative fatigue and anxiety.

Although breast pain was scored significantly higher by the patients in the hypnosis arm, this difference in pain level did not result at the clinical level in a higher consumption of ketamine and morphine in the PACU for these patients. Indeed, patients had similar need for rescue analgesia and pain relief in the PACU in the 2 arms. A 2014 meta-analysis by Kekecs et al^14^ showed that suggestive or hypnosis techniques reduced anxiety but did not significantly affect postoperative analgesic consumption. Furthermore, in the present study, an observed difference in breast pain was significant in the PACU in favor of the control arm but was not found for later time points.

There may be 2 reasons for our results herein. First, patients in the hypnosis arm received lower doses of the intraoperative anesthetic drugs propofol and sufentanil, which may explain the lower pain levels in the control arm. This may be linked to the statistically significant reduced PACU length of stay for patients in the hypnosis arm (median, 60 minutes [range, 20-290 minutes] in the control arm vs 46 minutes [range, 5-100 minutes] in the hypnosis arm; *P* = .002). Also, fewer patients received lidocaine in the control arm than in the hypnosis arm. This may have affected postoperative pain perception, together with the fact that more patients received PONV prophylaxis in the control arm, which may have resulted in less pain compared with the hypnosis arm. Second, the choice of surgery may also explain our results. Patients underwent minor breast cancer surgery, an intervention that induced limited postoperative pain because of a minimally invasive surgical technique (tumorectomy). We used a multimodal analgesic strategy (ie, a combination of several analgesic drugs injected before the end of anesthesia), which is the standard of care for pain control in these patients. In both study arms, patients received intravenous acetaminophen, ketoprofen, and ketamine. This strategy enabled effective pain relief by our teams for all patients. Indeed, patients in the control arm reported a low median pain intensity in the PACU (2 [range, 0-6] for breast pain and 0 [range, 0-4] for general pain). Reducing pain even further with hypnosis may have been an unreasonable goal because of these low pain levels.

The randomized clinical trial by Montgomery et al^12^ included 200 patients scheduled for breast surgery. Patients in the hypnosis arm underwent a 15-minute hypnosis session conducted by a psychologist. In that study, patients were not blinded to their study arm assignment, and the effectiveness of blinding of the research and clinical staff was not formally assessed. Blinding regarding treatment assignment usually gives stronger evidence of treatment efficacy than studies of unblinded design. When the treatment is hypnosis, blinding may decrease the potential for hypnotic suggestion and thus a positive effect of hypnosis. Montgomery et al^12^ reported a lower pain intensity in the PACU in the hypnosis arm compared with the control arm (mean VAS score [range, 0-100], 22.43 vs 47.83; *P* < .001). In that study, data were not available on pain reduction in the days after surgery.

Regarding air management herein, we found a significant difference between the 2 study arms. A noninvasive laryngeal mask was used more frequently for patients in the hypnosis arm (53.5% [38 of 71] of control patients vs 70.1% [54 of 77] of hypnosis patients, *P* = .04). This suggests that hypnosis may improve the quality of anesthesia induction and may allow better drug titration.

In our study, we assessed fatigue, comfort/well-being, and anxiety immediately before PACU discharge, at patient discharge (on the evening of surgery), and on the day after surgery. Only fatigue was found to be significantly lower among patients in the hypnosis arm on the evening of surgery compared with patients in the control arm. We performed a subgroup exploratory analysis that took into account perception of hypnosis by the patients. Indeed, 25.0% (14 of 56) of patients in the control arm thought that they had received hypnosis, while only standard welcoming and patient care had been used by their staff members, who were not trained in hypnosis techniques. This emphasizes the difficulty in analyzing the specific and intrinsic effect of hypnosis vs a putative placebo effect. The subgroup analysis showed significantly lower postoperative fatigue and anxiety levels and better comfort/well-being in the perceived hypnosis subgroup compared with the no perceived hypnosis subgroup. To our knowledge, this is the first study to report such an effect of hypnosis on postoperative fatigue. In oncology, cancer-related fatigue is a frequently present and disabling symptom, which often influences patient quality of life^20,21^ and for which no treatment is available. Therefore, hypnosis may mitigate postoperative fatigue in patients already experiencing cancer-related fatigue. However, no such effect was found 7 days after surgery in our study. An intermediate hypnosis session 3 days after surgery may extend the duration of the benefit of hypnosis on postoperative fatigue. Further studies could assess the effect of hypnosis on fatigue as a primary end point in patients undergoing oncologic surgery or surgery for other diseases. Studies assessing anxiety will also be relevant, with a previous study^22^ showing that nonpharmacological techniques, such as auricular acupuncture, can reduce dental anxiety to the same extent as intranasal midazolam hydrochloride.

Patient satisfaction regarding anesthesia care and global management (range, 0-10) was higher in the hypnosis arm (mean [SD], 8.9 [1.5] in the control arm vs 9.5 [1.1] in the hypnosis arm; *P* = .02]). A previous study^7^ showed that premedication with benzodiazepines did not improve the perioperative experience of patients when it was correlated with adverse effects, such as late extubation or a low early cognitive recovery rate, effects that have not been shown after hypnosis. Further studies are needed to objectivize and explain this higher global satisfaction and evaluate its causes, including simple attention and care linked to hypnosis or hypnosis-induced hormonal mediation influencing patient well-being.

Although not analyzed in our study, it may be possible that hypnosis has an effect on the operating staff members. In the hypnosis arm, patient preparation was performed with extra courtesy and in a low-noise and relaxed environment, which if studied may reveal a benefit on the whole team. Regarding the quality of life at work, it may be relevant to study this aspect and the effect of hypnosis on surgeons, nurses, and anesthesiologists in further studies.^23^

### Limitations

Our study has some limitations. As already discussed, the choice of surgery that induced limited postoperative pain is a major limitation and contributed to the negative primary end point of our study. Blinding may have decreased the effect of hypnosis because of hypnotic suggestion. Also, participation in the study may have caused a behavioral change among the medical and paramedical teams in the global care and management of patients, especially for patients in the control arm, who may have been cared for with more empathy than usual care. This may also have introduced a bias and decreased the difference in treatment group perception between the 2 arms and thus the effect of hypnosis in the hypnosis arm. Although patients in the control arm were prepared for surgery by staff not trained in hypnosis, a positive effect in addressing and caring for the patients makes it a limitation of the study.

## Conclusions

Our study shows no benefit of a short perioperative hypnosis session on postoperative pain in women eligible for minor breast cancer surgery. However, hypnosis seems to have other benefits regarding fatigue and anxiety, especially in patients who thought that they received hypnosis. Patient satisfaction is also improved with hypnosis. Further studies are needed to objectivize the benefit of hypnosis in this population.

## Notes

Supplement. **Trial Protocol**
