# Supplementary material for: Effects of a Hypnosis Session Before General Anesthesia on Postoperative Outcomes in Patients Who Underwent Minor Breast Cancer Surgery: The HYPNOSEIN Randomized Clinical Trial
Source: JAMA Netw Open. 2018 Aug 17;1(4):e181164. doi: 10.1001/jamanetworkopen.2018.1164 (PMC6324272; doi:10.1001/jamanetworkopen.2018.1164)
Supplement: Supplement. — Trial Protocol [file jamanetwopen-1-e181164-s001.pdf]

Added conversational hypnosis reduced general anesthesia side effects for day case breast surgery : A prospective randomized clinical trial

HYPNOSEIN

EudraCT Nº: 2014-A00681-46 **Date: June 3. 2014**
Study Nº: ICM-URC-2014/30 **Version Nº: version 1.1 (original protocol)**

**Sponsor**	**ICM**<br>Institut régional du Cancer de Montpellier<br>208, rue des Apothicaires<br>34298 Montpellier Cedex 5 - France<br>Phone: +33 (0) 4 67 61 31 02<br>Fax: +33 (0) 4 67 41 30 23<br>http://www.icm.unicancer.fr/
**Coordinator**	**Dr Jibba AMRAOUI**<br>**Anesthesia – Intensive Care Unit**<br>Institut régional du Cancer de Montpellier<br>Phone: +33 (0) 4 67 61 30 81<br>Fax: +33 (0) 4 67 61 00 00<br>Jibba.Amraoui@icm.unicancer.fr
**Clinical Research Associate**	**Melle Chloé JANISZEWSKI**<br>Institut régional du Cancer de Montpellier<br>Clinical and Translational Research Department<br>Phone: +33 (0) 4 67 61 23 08<br>Fax: +33 (0) 4 67 61 37 91<br>Chloe.janisweski@icm.unicancer.fr
**Biostatistician**	**Mme Marta JARLIER**<br>Institut régional du Cancer de Montpellier<br>Methodology and Data Management Center<br>Phone : +33 (0) 4 67 61 45 40<br>Fax: +33 (0) 4 67 61 37 18<br>marta.jarlier@icm.unicancer.fr

Ethics committee approval (CPP Sud Méditerranée III): 02/07/2014

Competent Authority approval ANSM: B140937-31

. Clause de confidentialité

Ce document contient des informations qui sont la propriété de l'Institut régional du Cancer Montpellier (ICM) et qui vous sont confiées à titre confidentiel pour être examinées par vous-même, votre équipe, et les Autorités administratives. Les informations contenues dans ce document ne doivent pas être communiquées à des tiers sans l'autorisation écrite préalable de l'ICM, à l'exception des éléments nécessaires à l'obtention du consentement éclairé des personnes qui pourraient se prêter à la recherche.

PROTOCOL SIGNATURE PAGE

Added conversational hypnosis reduced general anesthesia side effects for day case breast surgery: a prospective randomized clinical trial

Bénéfices attendus d'une hypnose médicale conversationnelle lors de l'induction anesthésique en chirurgie mammaire: Essai prospectif randomisé en simple aveugle

HYPNOSEIN

Writing Committee: Jibba Amraoui Jean-Pierre Bleuse; Chakib Sari; Marta Jarlier; Chloe Janiszewski.

**Sponsor**	**ICM**	**Date (dd-mm-yy)**	**Signature**
Managing Director	Pr. Jacques DOMERGUE		
Head of the Clinical and Translational Research Department	Dr. Jean-Pierre BLEUSE	24/04/2014	
**Titles**	**Name**	**Date (dd-mm-yy)**	**Signature**
Principal Investigator	Dr Jibba AMRAOUI		
Clinical Research Physician	Dr Chakib SARI		
Clinical Research Associate	Chloe JANISZEWSKI		
Biostatistician	Marta JARLIER		

Compliance statement

I undersigned Dr. ....

I have read the protocol, and agree to conduct the present clinical trial in accordance with applicable European Regulatory requirements, the Declaration of Helsinki and the principles of Good Clinical Practice Guidelines.

I agree:

- To obtain freely given written informed consent from each patient after she/he has been informed of all aspects of the trial relevant to his/her decision to participate
- To declare all serious adverse events within 24 hours after having been informed of the event
- To respect inclusion and non-inclusion criteria as well as the dates of onset and end of the study
- To fully complete all sections of the case report form (CRF)
- To provide any clarification or correction related to the CRF
- To permit regular monitoring and auditing by the Sponsor
- To archive and store all trial-related documents for a 15-year period

Investigator's agreement:

Date: **Signature:**

14

SYNOPSIS

**TITLE**	**Added conversational hypnosis reduced general anesthesia side effects for day case breast surgery: a prospective randomized phase III clinical trial.**<br><br>**Bénéfices attendus d'une hypnose médicale conversationnelle lors de l'induction anesthésique en chirurgie mammaire: Essai prospectif randomisé**
**PROTOCOL CODES**	**Acronym** : HYPNOSEIN<br>**Study number** : ICM URC-2014/30<br>**EudraCT number**: 2014-A00681-46
**STUDY DESIGN**	Prospective randomized multicenter Phase III single-blinded study comparing 2 admission/care techniques in operating room for patients eligible for minor cancer surgery.
**STUDY SPONSOR**	**ICM (Institut régional du Cancer de Montpellier)**<br>Clinical and Translational Research Department<br>208 rue des Apothicaires<br>34298 Montpellier Cedex 05 – France<br>Contact Name : Dr Jean-Pierre BLEUSE<br>Phone: +33 (0) 4 67 61 31 02<br>E-mail: jean-pierre.bleuse@icm.unicancer.fr
**STUDY COORDINATOR**	**Dr Jibba AMRAOUI**<br>ICM (Institut régional du Cancer de Montpellier)<br>Anesthesia-Reanimation Department<br>208 rue des Apothicaires<br>34298 Montpellier Cedex 05 – France<br>Phone: +33 (0) 4 67 61 30 81<br>E-mail: jibba.amraoui@icm.unicancer.fr
**STUDY PERIOD**	Enrolment start date: 09/2014<br>Enrolment stop date: 03/2015<br>Planned enrolment duration: 18 months<br>Mean duration of patient follow-up : 1 month
**EXPECTED NUMBER OF PATIENTS**	150 patients (75 pts per arm) were expected to be enrolled in this trial.
**EXPECTED NUMBER OF SITES**	5 centers :<br>• ICM – Montpellier<br>• CHU - Montpellier<br>• Clinique Saint Grégoire – Rennes<br>• CHU Carémeau – Nîmes<br>• Institut Paoli Calmette - Marseille
**BACKGROUND AND RATIONALE**	Breast cancer is the most frequent cancer in women in France [1]. Surgery is, to date, the key treatment for this disease [2]. During care in the operating room, patient arrival is an anxiety-inducing moment, because breast cancer often affects young women, with light or non-existent medical history, taken in a "hostile" environment: noisy, cold, austere, with unknown care pathway … before a surgery for a cancer which is often psychologically-difficult to cope with.

	For all these reasons, premedications using anxiolytiques (benzodiazepin-like) are usually given to decrease patients' anxiety. However, alternative non-pharmacological techniques have shown efficacy in decreasing perioperative pain and anxiety. Among these techniques, sophrology, reinsurance, respiratory techniques and medical hypnosis are among the preferred techniques.<br><br>Regarding hypnosis techniques, many studies have shown that perioperative hypnosis could decrease anesthetic drugs consumption, postoperative pain, length of stay in postoperative care unit (PACU) and numerous postoperative adverse events: nausea, vomiting (PONV), pain, etc…<br><br>Among the different hypnosis techniques proposed to patients in perioperative setting, medical conversational hypnosis consists in supporting the patient as soon as her admission in the operating room to general anesthetic induction. This technique aims at suggesting an agreeable and non-anxietic environment. In a preliminary study in 20 patients [5], which was then confirmed with a randomized trial in 200 patients [11], the authors found that conversational hypnosis, performed by a clinician psychologist 15 minutes before arrival of the patients in the operating room, was associated with a decrease of the postoperative adverse events, mean (CI 95%): PONV (18.9: 12-24), postoperative pain (25: 17-33), asthenia (24: 16-32), discomfort (20: 12-28), emotional charge (24: 18-31).<br><br>However, to date, and to our knowledge, no randomized trial has shown interest and benefit of a conversational hypnosis performed by an anesthesiologist during anesthetic induction.<br><br>We propose in the context of the HYPNOSEIN study to compare perioperative conversational medical hypnosis (hypnosis group) performed by a trained anesthesiologist to a control group with no hypnosis session.
**ELIGIBILITY CRITERIA**	**<u>Inclusion criteria</u>**<br>• Female > 18 years<br>• Patient with ASA* score 1 ,2, 3<br>• Minor Unilateral breast surgery indication (cancer tumorectomy, , tumorectomy with limited axillary node dissection,<br>• Day case surgery (ambulatory surgery – living Day0-Day1)<br>• General anesthesia required<br>• Written informed consent<br>• French medical benefit<br>**<u>Exclusion criteria</u>**<br>• Age < 18 years<br>• Patient with ASA score > 4;<br>• Body mass index < 15 or 45kg/T²;<br>• Major Surgery indication : mastectomy, bilateral surgery, full axillary dissection, major breast reconstruction, lumpectomy<br>• Patient refusing hypnosis<br>• Patient with previous surgery with hypnosis<br>• Psychic or mental Disorders<br>• Chronic pain<br>• Opiate therapeutic > 3 months<br>• Not ability to speak and read French language<br>• Deaf and dumb patient

	• Under guardianship patient or guardianship<br>*see appendix
**TREATMENT MODALITIES**	Patients randomly assigned the day of surgery in one of the two groups :<br>***Hypnosis group**: the conversational hypnosis (10-15 min) is standardized and performed just before intravenous general anesthesia induction in the operative room.<br>***Control group**: no special preparation before intravenous general anesthesia induction in the operative room.<br><br>For the groups, preoperative preparation and postoperative care, including analgesia, are similar and standard<br>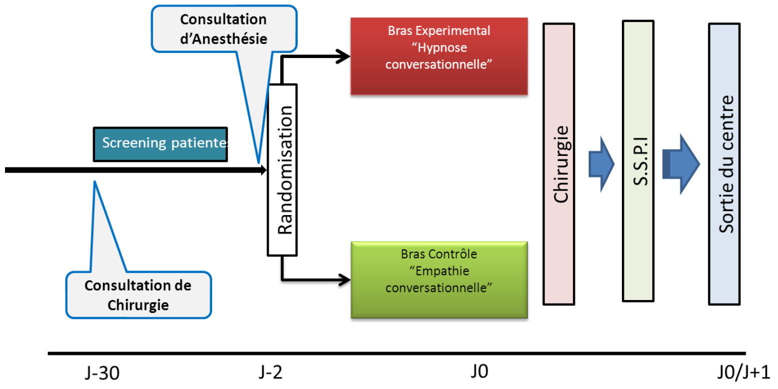
**OBJECTIVE**	Evaluate the impact of Added conversational hypnosis on reduced general anesthesia side effects (pain intensity) for day case breast surgery.
**PRIMARY ENDPOINT**	Reduction of the pain adverse postoperative outcome :<br>Using Visual analogic scale (VAS > 3/10)
**SECONDARY ENDPOINT**	**VAS evaluation of :**<br>- Post-surgery nausea and vomiting<br>- Fatigue (> 3/10)<br>- Discomfort (> 3/10)<br>- Emotional upset (3/10)<br>**Concomitant medication:**<br>- Used and dosage of antiemetic's<br>- Analgesic consumption<br>- Failed day case surgery<br>- Clinical times : operating room, post care unit
**STATISTICAL CONSIDERATION RANDOMIZATION**	The proposed study is an interventional multicenter randomized phase III controlled trial. Patients will be randomly allocated (1:1) to receive either the "conversational hypnosis" versus standard conversation. Randomization will be stratified according to Center,<br>Sample size calculation is based on the difference of at least 2 units between the 2 groups in term of Pain severity index (EVA). **150 patients (75 patients /arm**) were planned to be included.<br><br>**Planned number of subjects**<br>The necessary number of subjects was calculated according to the primary endpoint: pain severity measured with a 0 to 10 visual analog scale. The sample size calculation was based on a difference of at least 2

units on the VAS between the two groups in terms of pain severity. To detect such a difference, with an σ =3.5, a bilateral risk α=5% and a power of 90% (β=0.10), 66 patients per group are required. Considering 10% of non-evaluable patients, **a total of 150 patients, 75 per group, are planned.**

Calculations were made using the « Sample Size Tables for Clinical Studies Program » (Machin D, Campbell, Beng Tan S, Huey Tan S. Sample size table for clinical studies. Wiley-Blackwell. Comparing two independent groups for continuous data p 47 - Equation 5.2)

Statistical analyses

A descriptive analysis per group will be performed. The analyses will be performed on an intention-to-treat basis. Data will be described by treatment group. Continuous variables will be described using means with standard deviations, medians with interquartiles (IQ) according to their distribution. For categorical variables, frequencies and percentages will be computed.

It will be checked that the baseline characteristics are well-balanced between the two groups, and that they are thus comparable.

Efficacy of conversational hypnosis will be assessed comparing the pain severity score of the two groups (bilateral t-test, means and 95% confidence intervals).

A similar analysis will be performed using the Student's t-test or the Kruskal-Wallis test to compare the postoperative side-effects measured with a visual analogic scale (discomfort, fatigue, emotional upset) and for all quantitative variables among the secondary endpoints.

Standardized values (Δ/σ) will also be presented for each side-effect measured using a VAS.

The qualitative secondary endpoints will be compared between the two arms using a Chi-2 test or a Fisher exact test.

The analyses will be performed using the Stata v13 software after approval of the statistical analysis plan.

TABLE DES MATIERES

1. RATIONNEL ET JUSTIFICATION DE L'ETUDE..................
2. OBJECTIFS DE LA RECHERCHE..10
   - 2.1. Objectif principal..
   - 2.2. Objectifs secondaires..10
3. MÉTHODE DE LA RECHERCHE..
4. DESCRIPTION DE LA TECHNIQUE HYPNOTIQUE ....
5. CRITERES D'ELIGIBILITE...
   - 5.1. Critères d'inclusion :...12
   - 5.2. Critères de non inclusion :...12
6. DEROULEMENT DE LA RECHERCHE ....
   - 6.1. Récapitulatif du suivi patient ....
   - 6.2. Calendrier prévisionnel de l'étude ....
   - 6.3. Information et consentement..13
   - 6.4. Délai de réflexion et recueil du consentement..14
   - 6.5. Randomisation 14
   - 6.6. Admission au bloc opératoire et déroulement de l'anesthésie.......................................14
   - 6.7. Sortie du Bloc opératoire et Admission en SSPI 15
   - 6.8. Critères d'évaluation...
   - 6.9. Critère de jugement : ....
7. METHODOLOGIE STATISTIQUE ....
8. SECURITE DES PATIENTS ....
9. DROITS D'ACCES AUX DONNÉES ET DOCUMENTS SOURCE....................................20
   - 9.1. Accès aux données...
   - 9.2. Données sources ....
   - 9.3. Confidentialité des données ....
10. ASPECTS ÉTHIQUES ET RÉGLEMENTAIRES ....
    - 10.1. Responsabilités des investigateurs..21
    - 10.2. Information des patients 21
    - 10.3. Consentement des patients ....
11. ADMINISTRATION ET GESTION DE L'ÉTUDE....................
    - 11.1. Traitement des fiches recueillies...
    - 11.2. Dossiers des patients 23
    - 11.3. Classeur investigateur et archivage.......................................
    - 11.4. Monitorage, assurance qualité et inspections par les autorités
    - 11.5. Traitement des données relatives à la recherche...23
    - 11.6. Amendements au protocole de l'étude 23
12. ASSURANCE 24
13. PUBLICATION ET AUTEURS 24
14. RÉFÉRENCES BIBLIOGRAPHIQUES..25
15. ANNEXES…………………………………………………………………………..……31

LISTE DES ABREVIATIONS

AIVOC	Anesthésie Intra-Veineuse à Objectif de Concentration
ARC	Attaché de Recherche Clinique
ASA / PSC (score)	American Society of Anesthesiologists / Physical Status Score
CCTIRS	Comité Consultatif sur le Traitement de l'Information en matière de Recherche dans le domaine de la Santé
CPP	Comité de Protection des Personnes
DPO	Douleur Post-Opératoire
IADE	Infirmier Anesthésiste Diplômé d'Etat
ICM	Institut régional du Cancer de Montpellier
MAR	Médecin Anesthésiste-Réanimateur
NVPO	Nausées et Vomissements Post-Opératoire
SSPI	Salle de Surveillance Post-Interventionnelle
VAS	Visual Analogic Scale

1. BACKGROUND AND STUDY RATIONALE

Breast cancer is the most frequent cancer in women in France [1]. Surgery is, to date, the key treatment for this disease [2]. During care in the operating room, patient arrival is an anxiety-inducing moment, because breast cancer often affects young women, with light or non-existent medical history, taken in a "hostile" environment: noisy, cold, austere, with unknown care pathway … Breast cancer surgery is a painful moment, associated with cancer disease materialization. It is the time of a sudden awareness of the disease.

For all these reasons, premedications using anxiolytiques (benzodiazepin-like) are usually given to decrease patients' anxiety [3][4]. However, alternative non-pharmacological techniques have shown efficacy in decreasing perioperative pain and anxiety [5][6]. Among these techniques, sophrology, reinsurance, respiratory techniques and medical hypnosis are among the preferred techniques [5][6].

Regarding hypnosis techniques, many studies have shown that perioperative hypnosis could decrease anesthetic drugs consumption, postoperative pain, length of stay in postoperative care unit (PACU) and numerous postoperative adverse events: nausea, vomiting (PONV), pain … [5][6][7][8][9][10].

A study published in 1997 showed that perioperative hypnosis significantly decreased postoperative nausea and vomiting (39% *versus* 68% in the control group) [6]. A significant decrease of the emotional feeling score (16.5 *versus* 38.2, $p < 0.0001$, d = .85), the depression score (6.6 *versus* 19.9, $p < 0.02$, d = .67), the anxiety SV-POMS score (10.0 *versus* 5.0, $p < 0.0001$,d = 0.85) and the global anxiety score (75.7 *versus* 54.2, $p < 0.001$, d = -0.76) was reported [9].

Lang et al. [10] reported results in accordance with a reduction of anxiety and pain scores, which were reduced in the empathy and hypnosis groups *versus* the control groups:

- Anxiety: standard group (logit slope=0.18, p<0.001), empathy group (slope=-0.04, p=0.45), hypnosis group (slope=-0.27, p<0.001).

- Pain (logit slopes: standard care=0.53, empathy=0.37, hypnosis=0.34; all p<0.001).

In a surprising manner, a meta-analysis published in 2002 [11] and randomized trials [12] [13] report the clinical benefit of hypnosis in the treatment of breast cancer as adjuvant treatment.

Among the different hypnosis techniques proposed to patients in perioperative setting, medical conversational hypnosis consists in supporting the patient as soon as her admission in the operating room to general anesthetic induction. This technique aims at suggesting an agreeable and non-anxietic environment. In a preliminary study in 20 patients [5], which was then confirmed with a randomized trial in 200 patients [11], the authors found that conversational hypnosis, performed by a clinician psychologist 15 minutes before arrival of the patients in the operating room, was associated with a decrease of the postoperative adverse events, mean (CI 95%): PONV (18.9: 12-24), postoperative pain (25: 17-33), asthenia (24: 16-32), discomfort (20: 12-28), emotional charge (24: 18-31).

However, to date, and to our knowledge, no randomized trial has shown interest and benefit of a conversational hypnosis performed by an anesthesiologist during anesthetic induction.

Based on our clinical experience and on literature data [5] [13], we propose in the context of the HYPNOSEIN study to compare conversational hypnosis (hypnosis group) performed preoperatively by an anesthesiologist to no conversational hypnosis (control group). The primary objective was to evaluate the

effect of this patient support in terms of reduction of the major postoperative adverse events, especially pain but also anxiety, comfort, nausea and vomiting, and postoperative fatigue.

2. OBJECTIVES

2.1. Primary objective

To evaluate the impact of added conversational hypnosis performed by a medical anesthesiologist just before anesthetic induction on reduced general anesthesia side effects (pain intensity) for daycase breast surgery.

2.2. Secondary objectives

To evaluate the impact of conversational hypnosis on:

- Main postoperative adverse effects:
    - Nausea and vomiting
    - Comfort and well-being
    - Fatigue
    - Anxiety.

- Medical care and medical intake:
    - Analgesic drug consumption including morphinic drugs
    - Antiemetics use
    - Anxiolytics use (such as Benzodiazépine)

- Length of stay in postoperative care unit (PACU)

3. METHODOLOGY

3.1. Study design and groups

The present study is a prospective randomized multicenter Phase III single-blinded trial comparing two admission/care techniques in operating room for patients eligible for minor cancer surgery.

The patient will not know, before her admission, which technique will be used for admission in the operating room. The caregiver in charge of assessing outcomes will not know which technique was used.

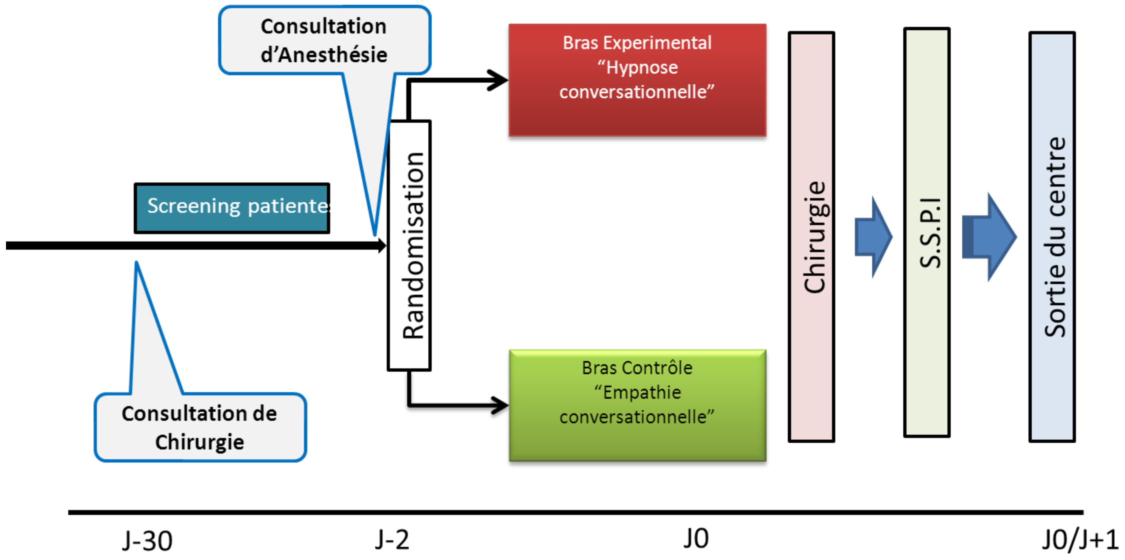

Hypnosis group: will use conversational hypnosis as communication technique with the patient during her admission in the operating room until the end of the anesthetic induction. <u>Conversational hypnosis will be performed solely by a medical anesthesiologist trained to the technique.</u>

Control group: will use the usual empathic communicating "technique" for admission in the operating room until the end of the anesthetic induction. <u>The usual empathic admission will be performed only by medical anesthesiologists not trained to the conversational hypnosis technique.</u>

4. HYPNOSIS TECHNIQUE

4.1. Conversational hypnosis technique for the hypnosis group:

A standardized conversational hypnosis technique will be used. Its objective will be to enhance comfort and well-being of the patient using various techniques. Themes will be proposed to each patient. The choice of a safe place or leisure activity will be done with the patient, and it will be personalized for each patient No theme will be imposed by the medical anesthesiologist, and the patient will be free to follow or not the anesthesiologist on the chosen theme. In case a patient asks to stop communication, the anesthesiologist will have to do so.

It is recommended that all teams use sensoriality language and paraverbal techniques (slow voice, low

tone). The duration of this communication and induction will be reported and will have to be equals or shorter than 15 minutes, except in case of technical problem. During the hypnosis session, only the anesthesiologist talked with the patient in order to individualize the session. The anesthesiologist will choose him/herself the best moment to perfom anesthetic induction.

Conversational hypnosis will be performed solely by a medical anesthesiologist trained to the technique. Only physicians who were trained to hypnosis (hypnosis university or non-university training) and who practiced hypnosis for 1 year or more.

4.2. Usual care for the control group

The caretakers will behave exactly "as usual" with usual empathy and care for patients in the control group. All caretakers who have had a training session on hypnosis or on communication in care will not be allowed communication with the patients of the control group, to avoid any biais. The anesthesiologist or anesthetic nurse performing anesthesia for patients in the control group should not have followed a hypnosis training. If these criteria are difficult to follow, caretakers will communicate only with empathy for patients in the control group. Musicotherapy in the OR will not be allowed, if usually performed.

5. ELIGIBILITY CRITERIA

5.1. Inclusion criteria

Patients were included in the study if they were

- women aged more than 18 years
- ASA (American Society of Anesthesiologists) < 4 (1, 2 or 3)
- patients with minor unilateral breast cancer surgery indication: cancer tumorectomy or tumorectomy with limited axillary node dissection.
- patients scheduled for day-case surgery (ambulatory, discharge on the same day or day +1)
- patients who gave their written informed consent for participating in the study
- patients with social security insurance

5.2. Non- inclusion criteria

- patients aged < 18 years

- ASA (American Society of Anesthesiologists) score ≥ 4

- body mass index <15 or > 45 kg/T^2

- indication for major surgery: mastectomy, bilateral surgery, full axillary dissection, major breast reconstruction, lumpectomy, previous duration of surgery > 2h

- patients refusing hypnosis or who had undergone previous surgery with hypnosis,

- patients with psychic or mental disorders, chronic pain or opiate therapeutic > 3 months

- patients not understanding French, deaf and/or mute

- patients under guardianship or curatorship

6. CLINICAL EVALUATION AND STUDY PROCEDURES

6.1. Patient follow-up

	D-30 to D-2	D0	D1	D7	D30
Information	X				
Informed consent	X				
Randomisation	X				
Pain assessment (VAS)		X			
Conversational hypnosis		X			
Anesthesia / Surgery		X			
Pain assessment (VAS) at PACU discharge		X	(X) Si sortie J1	X	
Safety follow-up and assessment of outcomes		X	(X) Si sortie J1	X	X

*D0 : Day of surgery

6.2. Study planning

First inclusion: 09. 2014

Inclusion period: 18 mois

Last inclusion: 03. 2015

End of follow-up period: 06.2015

Follow-up period: Maximum 1 month

6.3. Information and consent

The consent form of the study will be given to the patient by the surgical team at the date of the surgery plannigication (D-30 to D-7), as well as during anesthesia consultation at least 48h before surgery (usually D-7 to D-2).

The anesthesiologist will check the inclusion and non-inclusion criteria, inform the patient of the study details, give the patient the consent form and validate the patient's consent.

6.4. Reflection time and consent

A reflection time will be given to the patient between the anesthesia consultation and surgery, at least 48h. The patient's decision to participate or not in the study will have to be known at the latest at her admission for surgery. Dated and signed consent will be collected at that time.

6.5. Randomisation

Randomization will be centralised by the sponsor at the Biostatistic Unité of the Montpellier Cancer Institute (ICM Val d'Aurelle, Montpellier). After signed consent and medical validation of the initial check-up results, eligible patients will be randomized.

Patient randomisation will be performed at the end of the anesthesia consultation. The inclusion form will be completed and signed by the investigator (Appendix 1). The form will be sent by fax at the following:

Institut régional du Cancer de Montpellier
Unité de biostatistique
Phone: +33 (0) 4 67 61 45 40
Fax: +33 (0) 4 67 61 37 18

The anesthesilogist will be appointed to the patient after randomisation, depending on the allocated group.

6.6. Operating room admission and anesthesia

No premedication is allowed to be given to the patient, who will have fasted for 4h for liquids. If any premedication was given, it should be recorded.

At operating room admission, the two groups are to perform an oral questionnaire, the safety check-up (legal requirement from the Haute Autorité de Santé). Then :

- For the control group, physicians and all caregivers will behave "as usual"
- For the hypnosis group, patient will be welcomed by a specific anesthesiologist trained for hypnosis, who will start the hypnosis technique as soon as the patient is admitted in the operation room, and until anesthesia induction. Duration of the hypnosis session will be of maximum 15 minutes. This duration does not accounted for the check-up list. If, for any reason, supplemental time is needed, it should be recorded, together with the reason (technical problem, agitated patient, etc…)

<u>L'induction anesthésique (habituelle ou recommandée) :</u>

- Propofol en AIVOC (cible 4 à 6), ou en bolus 2 à 2.5 mg/kg

- Sufentanil 0.15 à 0,25 µg/kg, curare si besoin. (à noter)

Entretien : par Propofol (AIVOC), ou halogenes

Contre- indication du protoxyde d'azote

Dispositif des voies aériennes

Intubation orotrachéale

ou

Masque laryngé.

Selon le choix du clinicien

Autres Médications (habituelle ou recommandée) :

ketamine : 0,15-0,20mg/kg (en absence de contre-indication)

Lidocaine : 1 mg/kg (en absence de contre -indication)

On notera les doses d'agents utilisés à l'induction et pour l'entretien.

Les antalgiques autorisés : Paracetamol, Profenid, Acupan (en absence de contre- indication).

le Tramadol en prophylactique n'est pas indiqué.

Prophylaxie anti nausée vomissement (NVPO) uniquement pour les patientes ayant un score APFEL (Annexe 4) > 2 :

Dexamethasone (1 à 4 mg) et Droleptan (0, 625 à 1,25 mg) par voie IV

Les cétrons sont injectés en SSPI si besoin.(à justifier)

All drugs used perioperatively will be standardized and will not differ in the two groups.

6.7. Operating room discharge and admission in PACU

At the end of surgery, the patient will be brought in PACU.

PACU discharge will be allowed when the ALDRETE score will be >12/14 (see Appendix 2).

Discharge from the center to home will be allowed:

- In outpatient surgery, after approval by the anesthesiologist or the surgeon. If the patient is not allowed the discharge, the reason should be recorded.
- In classic surgery on day 1. If the patient is not allowed the discharge, the reason should be recorded.

6.8. Evaluation criteria

Assessments of the following criteria will be performed by a caretaker or clinical research assistant, blinded regarding the technique undergone by the patients. Assessment will be made in PACU or ACU, and by phone calls at D1, D7 and D30.

The main characteristics measured are:
- Post-surgery nausea and vomiting, with a 0 to 10 visual analogic scale ("face scale", Appendix 6).
- Pain in PACU with a 0 to 10 visual analogic scale
- Pain-related discomfort with a 0 to 10 visual analogic scale ("face scale", Appendix 6).
- Comfort/well-being with a 0 to 10 visual analogic scale ("face scale", Appendix 6).
- Anxiety with a 0 to 10 visual analogic scale ("face scale", Appendix 6).
- Fatigue with a 0 to 10 visual analogic scale ("face scale", Appendix 6).

Other criteria are reported :
- Concomitant medication
- Used and dosage of antiemetic's
- Analgesic consumption
- Failed day case surgery
- Clinical times : operating room, post care unit

Also :
- Patients characteristics: age, sex, weight, height, previous treatments and previous surgeries, tabacco and alcohol comsumption, main treatments
- Surgical characteristics: intervention, surgical act, side of surgery, duration of surgery
- Anesthetic characteristics: anesthetic drugs used in perioperative context and duration of anesthesia
- Duration of stay in PACU
- Anti-emetics, morphinic, etc… compsumption,

6.9. Endpoints

Primary endpoint:

Reduction of the pain adverse postoperative outcome using a Visual analogic scale (VAS > 3/10). VAS pain score.

Secondary endpoints:

VAS evaluation of:
- Post-surgery nausea and vomiting (occurrence and score)
- Fatigue (> 3/10)
- Comfort (<7/10)
- Emotional upset (<7/10)

Concomitant medication:
- Used and dosage of antiemetic's
- Analgesic consumption
- Failed day case surgery
- Clinical times : operating room, post care unit

7. STATISTICAL CONSIDERATIONS

7.1. Sample size

The necessary number of subjects was calculated according to the primary endpoint: pain severity measured with a 0 to 10 visual analog scale. The sample size calculation was based on a difference of at least 2 units on the VAS between the two groups in terms of pain severity. To detect such a difference, with an σ =3.5, a bilateral risk α=5% and a power of 90% (β=0.10), 66 patients per group were required. Considering 10% of non-evaluable patients, a total of 150 patients, 75 per group, were planned.

Calculations were made using the « Sample Size Tables for Clinical Studies Program » (Machin D, Campbell, Beng Tan S, Huey Tan S. Sample size table for clinical studies. Wiley-Blackwell. Comparing two independent groups for continuous data p 47 - Equation 5.2)

7.2. Statistical analyses

A descriptive analysis per group will be performed. The analyses will be performed on an intention-to-treat basis. Data will be described by treatment group. Continuous variables will be described using means with standard deviations, medians with interquartiles (IQ) according to their distribution. For categorical variables, frequencies and percentages will be computed.

It will be checked that the baseline characteristics are well-balanced between the two groups, and that they are thus comparable.

Efficacy of conversational hypnosis will be assessed comparing the pain severity score of the two groups (bilateral t-test, means and 95% confidence intervals).

A similar analysis will be performed using the Student's t-test or the Kruskal-Wallis test to compare the postoperative side-effects measured with a visual analogic scale (discomfort, fatigue, emotional upset) and for all quantitative variables among the secondary endpoints.

Standardized values (Δ/σ) will also be presented for each side-effect measured using a VAS.

The qualitative secondary endpoints will be compared between the two arms using a Chi-2 test or a Fisher exact test.

The analyses will be performed using the Stata v13 software after approval of the statistical analysis plan.

8. SAFETY

8.1. Adverse events

An Adverse Event (AE) is defined as any untoward medical occurrence in a subject or clinical investigation subject administered an investigational product and which does not necessarily have a causal relationship with this product". An AE can therefore be any unfavorable and unintended sign (for example: an abnormal laboratory finding), symptom, disease, or worsening of a pre-existing medical condition

temporally associated with the use of an investigational product, whether or not considered related to the investigational product.

8.2. Expected adverse events

Only Adverse Events (AEs) imputable to conversational hypnosis will be reported.

8.3. Serious Adverse Events (SAEs)

A Serious Adverse Event (SAE) is an adverse event which:

- results in death
- is life-threatening
- requires inpatient hospitalization (>24h) or prolongation of existing hospitalization,
- results in persistent or significant disability or incapacity
- is a congenital anomaly/birth defect, or
- medically relevant

For every SAE the Investigator and the Sponsor evaluate separately the possible causal relationship to the investigational product.

8.4. Severity criteria

The severity criteria should not be mistaken with the seriousness criteria which determine the conditions of notification. The severity or grade of adverse events is evaluated by the Investigator following the NCI-CTCAE classification version 4.0.

The CTCAE displays Grades 1 through 5 with unique clinical descriptions of severity for each AE based on this general guideline:

- Grade 1 = Mild; asymptomatic or mild symptoms; clinical or diagnostic observations only; intervention not indicated.
- Grade 2 = Moderate; minimal, local or noninvasive intervention indicated; limiting age-appropriate instrumental ADL (Any Day Life)*.
- Grade 3 = Severe or medically significant but not immediately life-threatening; hospitalization or prolongation of hospitalization indicated; disabling; limiting self-care ADL**.
- Grade 4 Life-threatening consequences; urgent intervention indicated.
- Grade 5 Death related to AE.

*Instrumental ADL refer to preparing meals, shopping for groceries or clothes, using the telephone, managing money, etc.

**Self-care ADL refer to bathing, dressing and undressing, feeding self, using the toilet, taking medications, and not bedridden.

8.5. SAE notification procedure

Every SAE, expected or unexpected, occurring during the study period and linked to conversational hypnosis practice should be notified to the Sponsor without any further delay, and within 24 working hours in all cases, using the "Serious Adverse Event Notification Form" (Appendix 4). This form should be completed following the completion instructions (Appendix 7), in English with only one diagnosis, and be faxed to the ICM Clinical Research Pharmacovigilance Unit.

Clinical Research Pharmacovigilance Unit
Mme Nadia Bensmail
ICM, Bât. A
208, rue des Apothicaires, 34298 Montpellier
Tel: +33 4 67 61 45 68 – Fax: +33 4 67 61 31 04

Every SAE occurring beyond the 28-day period after the withdrawal of the investigational product, judged by the Investigator to be related to the investigational product, should also be notified to the Sponsor in the same conditions as every other SAE.

The "Serious Adverse Event Notification Form" should be completed in English and only one diagnosis or one symptom (except for linked symptoms) should be reported to enable MedDRA coding. If several symptoms are documented in the source documents, only the main symptom will be reported as verbatim on the notification form.

After the initial notification, a follow-up report should be completed and faxed every time complementary information on the SAE becomes available. Finally, when the case is closed, a final report with the complete information should be completed and faxed to the Pharmacovigilance Unit (Fax: +33 4 67 61 31 04).

Complementary information or clarification could be requested by the Sponsor using Data Clarification Forms (DCFs). The Sponsor could also ask the site to send the anonymized medical records or laboratory findings corresponding to the SAE. The ICM, as the Sponsor of the trial, receives all SAE Notification Forms and evaluates the imputability and the expectedness of the SAEs. The declaration of eventual SUSARs and New Safety Issues to the competent authorities is delegated to UNICANCER, 101 rue de Tolbiac 75013 Paris (Tel: +33 1 44 23 04 04). UNICANCER submits the SUSARs and New Safety Issues within the required regulatory timelines to the European Medicines Agency (EMA) via EudraVigilance and to the competent national authorities and ethics committees (ANSM and CPP).

The risk-benefit balance of the study is evaluated continuously by the ICM Clinical Research Pharmacovigilance Unit and this risk-benefit balance will be discussed in the periodic safety reports. These reports will contain all required regulatory aspects and will be submitted to the competent authorities.

In the present study, all SAEs imputable to surgery, to cancer treatment or to any concomitant treatment will not be notified to the study Sponsor. Only the SAEs linked to the study procedures will be reported to the study Sponsor.

9. DROITS D'ACCES AUX DONNÉES ET DOCUMENTS SOURCE

9.1. Data

Data collected in this trial will be sent to the Coordinating Center in Montpellier for primary evaluation and follow-up.

All data obtained in the study described in the protocol will be recorded on CRF. The CRF for each subject will be presented in a folder. The CRF will be completed chronologically and updated regularly in order to reflect the most recent data on the subject included in the study.

Each CRF must be neatly filled in with a black-inked pen. For each page on which information is entered the subject number must be recorded. The registration form, the DLT form, the end of treatment form, the follow-up status form and the death report form must be dated and signed by an authorized Investigator.

Error must be corrected by drawing a single line through the incorrect entry and by writing the new value as close as possible to the original. The correction must then be initialed and dated by an authorized person.

Although subjects may be interviewed by a CRA, the Investigator must verify that all data entries are accurate and correct, including verification that the subject fulfils the criteria for entrance into the study before study medication is dispensed. Physical examinations have to be performed by a registered medical practitioner.

The End of Treatment Form must be completed for each subject either finishing the study or dropping out from it.

The Investigator will add to the subject trial file, after completion of the study, any relevant post-trial information brought to his attention. This information will be sent to the Sponsor within one year after ending the trial or more if the need arises.

Original forms and the first two copies of these forms are the property of the Sponsor.

9.2. Source document

Data for this study will be recorded via Case Report Forms (CRFs). Accurate and reliable data collection will be assured by verification and cross–check of the CRFs against the Investigator's records by the study monitor. It should be defined as source document verification.

9.3. Data confidentiality

According to the current regulation (articles L.1121-3 et R.5121-13 du code de la santé publique), all persons with access to confidential information will ensure confidentiality of all data (treatment, research,

persons and patients, especially patient ID, study results). They are all subject to professional and medical secrecy.

The present materials (protocol, CRF, investigator's brochure) contain confidential information. Except as may otherwise be agreed to in writing with the study monitor, the investigator agrees to hold such information in confidence, and not to disclose it to others (except where required by applicable laws and regulations). All information from this study (excluding data from informed consent) will be entered into a database by the sponsor in accordance with the French law,"Loi Informatique et Libertés" (art. 40, January 6, 1978) and with the European Directive 95/46/CE. All data will be anonymized (first letter of the first name and surname, inclusion number, center number).

The sponsor will be responsible for ensuring that all patients participating in the study have given their informed consent for individual access to their personal information.

10. ETHICAL AND REGULATORY CONSIDERATIONS

10.1. Regulatory and ethical compliance

It is mandatory that all considerations regarding the protection of human subjects be carried out in accordance with the protocol, Good Clinical Practices (GCP), ICH Guidelines, the ethical principles that have their origin in the Declaration of Helsinki, and all applicable regulatory requirements.

10.2. Responsibilities of the investigators

The investigator will be responsible for the conduct of the study trial according to the following texts and reglementation:

- Les recommandations de la "Déclaration d'Helsinki" révisée à Tokyo, Venise, Hong-Kong, Somerset West et Édimbourg les BPC de la Conférence Internationale d'Harmonisation (ICH-E6, 17/07/96)
- La loi de santé publique (n° 2004-806) du 9 août 2004 et le décret d'application n° 2006-477 du 26 avril 2006 relatifs aux recherches biomédicales,
- à la loi n° 2004-801 du 6 août 2004 relative à la protection des personnes physiques à l'égard des traitements de données à caractère personnel et modifiant la loi n° 78-17 du 6 janvier 1978 relative à l'informatique, aux fichiers et aux libertés,
- à la directive européenne (2001/20/CE) sur la conduite des essais cliniques.

All these texts remind that a written consent is to be given by all patients before their participation in the study.

10.3. Patients' information and consent

The "Declaration of Helsinki" recommends that consent should be obtained from each potential subject in biomedical research trials after the aims, methods, anticipated benefits, and potential hazards of the trial, and discomfort it may entail, are explained to the individual by the physician. The potential study subject should also be informed of his or her right to not participate or to withdraw from the trial at any time.

The patient should be told that material from her/his tumor will be stored and potentially used for additional studies not described in this protocol.

Informed consent for each subject will be obtained prior to initiating any trial procedures according to the current regulation (directive ICH E6, 1995).

The One copy of the informed consent must be given to each subject and one signed original copy must be retained in the investigator's trial records. The informed consent form must be available in the case of data audits.

If the subject is in a dependent relationship to the physician or gives consent under duress, the informed consent should be obtained by an independent physician. By signing this protocol, the investigator agrees to conduct the trial in accordance with the "Declaration of Helsinki".

10.3 Regulatory authority approvals

All protocols and the subject informed consent forms must have the approval of a properly constituted committee or committees responsible for approving clinical trials.

The study will be conducted in accordance with French regulation:

- les dispositions relatives à la recherche biomédicale du Code de la Santé publique, articles L1121-1 et suivants (loi de santé publique du 9 août 2004),
- les lois de Bioéthique,
- la loi informatique et libertés,
- la déclaration d'Helsinki,
- et les Bonnes Pratiques Cliniques.

11. STUDY ADMINISTRATION

The study data will be recorded directly by the identified and declared persons of each center, via the eCRF, and will be controlled and validated according to specific procedures.

At the end of the study and once all the eCRF data are validated, the investigator will log in and sign all the pages in order to validate the data entered for each patient.

The sponsor will create and send an electronic copy (PDF file) to the investigator. This copy must be printed and signed by the investigator, to be archived at the investigator's site.

11.1. Collected data

Case-report forms will be given by the Montpellier Cancer Institute (ICM Val d'Aurelle). Each CRF will be mentionning (among many):

- Surname (3 letters) and first name (3 letters) of the patient
- Date of birth, gender

CFRs must be filled in, using a black pen, in a readible or thorough manner. Any necessary correction/modification will be performed apparently and signed by the investigator, or a third party designated by the investigator, with date and time of the modification recorded. Mistakes should stay readable and cannot be erased with any erasing technique.

In case of missing data, coding to be used will be specified in the CRF.

11.2. Patient files

The investigator will be responsible for source data for each patient (paper or digital data).

11.3. Study monitoring

A Clinical Research Associate (CRA) will be appointed by the Sponsor to monitor this study.

CRA activities include: Site initiation visit to collect and distribute essential pre-study documents; to instruct the investigator and site personnel about the protocol, study procedures and expectations; to obtain investigator's assurance to comply with study requirements and GCP guidelines and to inform the investigator and appropriate study staff about study materials.

Monitoring visits : according to Good Clinical Guidelines, the study CRAs involved in the present study are fully instructed concerning confidentiality and able to perform any necessary control on informed consent and CRFs, including cross-checking clinical and laboratory data with the subject's file. All observations and findings should be verifiable. During monitoring visits, the Sponsor CRAs will:

- check and assess the progress of the study;
- review study data collected;
- conduct Source Document Verification (hospital files);
- identify any issue and address its resolution;

This will be done in order to verify that the:

- Data are authentic, accurate, and complete.
- Safety and rights of subjects are being protected;
- Study is conducted in accordance with the currently approved protocol (and any amendments), GCP and all applicable regulatory requirements.

The investigator agrees to allow the CRA direct access to all relevant documents and to allocate his/her time and the time of his/her staff to the CRA to discuss findings and any relevant issues.

11.4. Traitement des données relatives à la recherche

In accordance with the Decree on informatics and freedom of August 6th, 2004, the follows the reference methodology MR001 of the National Commission for Data Processing and Freedoms.

11.5. Amendements of the protocol

The sponsor alone is authorized modifying the protocol, in consultation with the trial coordinator.

In accordance with the Articles L.1123-9 and R.1123-35 of the French Public Health Code, any change occurring after the beginning of the research, having an impact on any aspect of the research, especially on protection of persons, including with regard to their safety, on the conditions for the validity of research, if

any, on the quality and safety of experimental drugs, on the interpretation of scientific documents which support the development of research or the modalities of conduct of this one.

A substantial modification request is sent by the sponsor to the CPP for an opinion. Upon receipt of the favorable opinion, the amended version of the protocol is then forwarded for information to the ANSM and forwarded to all investigators by the sponsor.

A non-substantial change to the protocol is a minor change or unrestricted clarification of the conduct of the test. These modifications will not be submitted to the competent authorities but will be subject to an agreement between the sponsor and the investigator and will be clearly documented in the follow-up file of the study and will be forwarded to the CPP for information.

12. ASSURANCE

12.1. Quality Assurance

Prior to the enrollment of any subject at a site, the investigator will review the protocol, investigator brochure, the procedure for obtaining informed consent, and procedures for reporting adverse events.

The investigator is required to retain subject identification codes for a minimum of 15 years after completion or discontinuation of the trial. The investigator is required to retain all subject files and source documents for the maximum period of time permitted by the hospital, institution, or private practice, but for not less than 10 years in order to meet international registration requirements.

12.2 Trial Insurance

As study Sponsor, the ICM has subscribed to an insurance against civil liability in accordance with applicable regulatory requirements of the Article L1121-10 of the French Public Health Code: SHAM – 18 rue Edouard Rochet - 69372 Lyon Cedex 08 (contract n° 140.474). Our insurance program covers all patients entered in this study within the European Union. This insurance program covers the sponsor, the investigators and all local hospital staff.

13. PUBLICATION AND AUTHORSHIP

All data issued from the trial are considered confidential at least until analysis and control by the sponsor, the principal investigator and the biostatistician of the study.

All publications, abstracts or oral presentation including results of the study should be submitted to the sponsor's approval.

The principal investigator of the study will be the corresponding author of the communication and will write the publication; he/she may ask for the help of the person he/she will appoint for the task.

REFERENCES

[1] Leclère B, Molinié F, Trétarre B, Stracci F, Daubisse-Marliac L, Colonna M. Trends in incidence of breast cancer among women under 40 in seven European countries: A GRELL cooperative study. Cancer Epidemiol 2013;37:544–549.

[2] Rouanet P. [Current position of breast reconstruction in oncology]. Gynecol Obstet Fertil 2002;30:985–993.

[3] Adam F, Bordenave L, Sessler DI, Chauvin M. Effects of a single 1200-mg preoperative dose of gabapentin on anxiety and memory. Ann Fr Anesth Reanim 2012;31:e223–227.

[4] Ismail SA, Mowafi HA. Melatonin provides anxiolysis, enhances analgesia, decreases intraocular pressure, and promotes better operating conditions during cataract surgery under topical anesthesia. Anesth Analg 2009;108:1146–1151.

[5] Eberhart LH, Döring HJ, Holzrichter P, Roscher R, Seeling W. Therapeutic suggestions given during neurolept-anaesthesia decrease post-operative nausea and vomiting. Eur J Anaesthesiol 1998;15:446–452.

[6] Enqvist B, Björklund C, Engman M, Jakobsson J. Preoperative hypnosis reduces postoperative vomiting after surgery of the breasts. A prospective, randomized and blinded study. Acta Anaesthesiol Scand 1997;41:1028–1032.

[7] Faymonville ME, Fissette J, Mambourg PH, Roediger L, Joris J, Lamy M. Hypnosis as adjunct therapy in conscious sedation for plastic surgery. Reg Anesth 1995;20:145–151.

[8] Lew MW, Kravits K, Garberoglio C, Williams AC. Use of preoperative hypnosis to reduce postoperative pain and anesthesia-related side effects. Int J Clin Exp Hypn 2011;59:406–423.

[9] Schnur JB, Bovbjerg DH, David D, Tatrow K, Goldfarb AB, Silverstein JH, Weltz CR, Montgomery GH. Hypnosis decreases presurgical distress in excisional breast biopsy patients. Anesth Analg 2008;106:440–444, table of contents.

[10] Lang EV, Berbaum KS, Faintuch S, Hatsiopoulou O, Halsey N, Li X, Berbaum ML, Laser E, Baum J. Adjunctive self-hypnotic relaxation for outpatient medical procedures: a prospective randomized trial with women undergoing large core breast biopsy. Pain 2006;126:155–164.

[11] Montgomery GH, David D, Winkel G, Silverstein JH, Bovbjerg DH. The effectiveness of adjunctive hypnosis with surgical patients: a meta-analysis. Anesth Analg 2002;94:1639–1645, table of contents.

[12] Spiegel D, Bloom JR. Group therapy and hypnosis reduce metastatic breast carcinoma pain. Psychosom Med 1983;45:333–339.

[13] Lang EV, Berbaum KS, Faintuch S, Hatsiopoulou O, Halsey N, Li X, Berbaum ML, Laser E, Baum J. Adjunctive self-hypnotic relaxation for outpatient medical procedures: a prospective randomized trial with women undergoing large core breast biopsy. Pain 2006;126:155–164.

14. ANNEXE

ANNEXE 1 : FICHE D'ENREGISTREMENT

ANNEXE2 : SCORE D'ALDRETE

ANNEXE3 : SCORE ASA

ANNEXE 4 : SCORE D'APFEL

ANNEXE 5 : DÉCLARATION D'HELSINKI

ANNEXE 6 : SCORE EVA PATIENT

ANNEXE 7 : QUESTIONNAIRE EVAN/APAIS

Biometrics unit
CTD labelled by INCa

Statistical Analysis Plan

PROTOCOL [ICM-URC-2014/30]
N° EudraCT : 2014-A00681-46

BENEFICES ATTENDUS D'UNE HYPNOSE MEDICALE CONVERSATIONNELLE LORS DE L'INDUCTION ANESTHESIQUE EN CHIRURGIE MAMMAIRE : ESSAI PROSPECTIF RANDOMISE DE PHASE III EN SIMPLE AVEUGLE.

HYPNOSEIN

Edition n° 1 of 21/10/2016

Sponsor : ICM
208, rue des Apothicaires
34298 Montpellier cedex 05

Coordinator : Dr Jibba AMRAOUI
Tel : 04.67.61.30.81
E-Mail : Jibba.Amraoui@icm.unicancer.fr

Biostatistician: Marta JARLIER / Julien FRAISSE
Tel : 04.67.61.45.57 / 30.30 – Fax : 04.67.61.37.18
E-Mail : marta.jarlier@icm.unicancer.fr
julien.fraisse@icm.unicancer.fr

**N° du Protocol :**	ICM-URC-2014/30
**Study Phase :**	Phase III
**Investigator:**	Dr Jibba AMRAOUI, ICM, Montpellier, France.
**Author (s):**	Marta JARLIER<br>N° tel : 04.67.61.45.57 / N° fax : 04.67.61.00.00<br>E-mail : marta.jarlier@icm.unicancer.fr<br>Date : 29/09/2016 Signature : 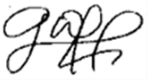
**Reviewer (s):**	Julien FRAISE<br>N° tel : 04.67.61.30.30 / N° fax : 04.67.61.37.18<br>E-mail : julien.fraisse@icm.unicancer.fr<br>Date : 16/12/2016 Signature : 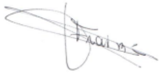
**Coordinator:**	Jibba AMRAOUI<br>N° tel : 04.67.61.30.81 / N° fax : 04.67.61.00.00<br>E-mail : jibba.amraoui@icm.unicancer.fr<br>Date : Signature :

**REVISION n°**	**Date**	**Author**	**Reason**
**1.0**	**16/12/2016**	**Marta Jarlier / Julien Fraisse**	**Primary endpoint analysis**

SOMMAIRE

ABREVIATIONS LIST **4**
1. SYNOPSIS **5**
2. ASSESMENT CRITERIA **10**
2.1 PRIMARY ENDPOINT 10
2.2 SECONDARY ENDPOINTS 10
3. PLANNED ANALYSES **11**
4. DEFINITION OF POPULATIONS **11**
5. MATERIAL AND METHODS **12**
5.1 DEFINITION AND CONVENTIONS 12
5.1.1 Conventions 12
5.1.2 Missing data 12
5.2 MATERIAL 13
5.2.1 Subjects disposition 13
5.2.2 Stratification factors 13
5.2.3 Baseline characteristics 13
5.2.4 Treatment administration 14
5.2.5 Toxicity evaluation 15
5.2.5.1 Adverse events 15
5.2.6 Efficacy evaluation 15
5.2.6.1 Primary endpoint 15
5.2.6.2 Secondary endpoints 16
5.2.7 Concomitant treatments 16
5.3 STATISTICAL METHODS 16
5.3.1 Descriptive statistics 16
5.3.1.1 Continuous variables 16
5.3.1.2 Categorical variables 16
5.3.2 Survival data 16
6. APPENDIX **17**
6.1 APPENDIX 1: LIST OF TABLES AND DATA LISTINGS 17
6.1.1 Baseline 17
6.1.2 Treatment 17
6.1.3 Toxicity evaluation 17
6.1.4 Efficacy 17
6.1.5 End of study 17

ABREVIATIONS LIST

CPP	Comité de Protection des Personnes
DC	Dose cumulée
DI	Dose Intensité
DIR	Dose Intensité Relative
DSMB	Data and Safety Monitoring Board
EI	Evènement Indésirable
EIG	Evènement Indésirable Grave
EORTC	European Organisation for Research and Treatment of Cancer
HR	Hazard Ratio
ICH	International Conference on Harmonisation
IC95%	Intervalle de Confiance 95%
IMC	Indice de Masse Corporelle
ITT	Intend To Treat = Intention de traiter
NCI-CTC	National Cancer Institute-Common Terminology Criteria
PAS	Plan d’Analyse Statistique
PFS	Progression Free Survival
PP	Per-Protocole
OS	Overall Survival
QLQ	Quality of life Questionnaire
SC	Surface Corporelle
SOC	System Organ Class
**Spécifiques à ce protocole :**	
MAR	Médecin Anesthésiste-Réanimateur
SSPI	Salle de Surveillance Post interventionnelle
NVPO	Nausée, Vomissement

The statistical analysis plan (SAP) describes the statistical analyzes to be carried out for the HYPNOSEIN study.

This SAP was written from the following documents:

- Original protocol containing all the amendments
- International Conference on Harmonisation (ICH) guideline E9 (Statistical Principles for Clinical Trials).

1. SYNOPSIS

**TITLE**	**Added conversational hypnosis reduced general anesthesia side effects for day case breast surgery: a prospective randomized phase III clinical trial.**<br><br>**Bénéfices attendus d'une hypnose médicale conversationnelle lors de l'induction anesthésique en chirurgie mammaire: Essai prospectif randomisé**
**PROTOCOL CODES**	**Acronym** : HYPNOSEIN<br>**Study number** : ICM URC-2014/30<br>**EudraCT number**: 2014-A00681-46
**STUDY DESIGN**	Prospective randomized multicenter Phase III single-blinded study comparing 2 admission/care techniques in operating room for patients eligible for minor cancer surgery.
**STUDY SPONSOR**	**ICM (Institut régional du Cancer de Montpellier)**<br>Clinical and Translational Research Department<br>208 rue des Apothicaires<br>34298 Montpellier Cedex 05 – France<br>Contact Name : Dr Jean-Pierre BLEUSE<br>Phone: +33 (0) 4 67 61 31 02<br>E-mail: jean-pierre.bleuse@icm.unicancer.fr
**STUDY COORDINATOR**	**Dr Jibba AMRAOUI**<br>ICM (Institut régional du Cancer de Montpellier)<br>Anesthesia-Reanimation Department<br>208 rue des Apothicaires<br>34298 Montpellier Cedex 05 – France<br>Phone: +33 (0) 4 67 61 30 81<br>E-mail: jibba.amraoui@icm.unicancer.fr
**STUDY PERIOD**	Enrolment start date: 09/2014<br>Enrolment stop date: 03/2015<br>Planned enrolment duration: 18 months<br>Mean duration of patient follow-up : 1 month
**EXPECTED NUMBER OF PATIENTS**	150 patients (75 pts per arm) were expected to be enrolled in this trial.

**EXPECTED NUMBER OF SITES**	5 centers :<br>• ICM – Montpellier<br>• CHU - Montpellier<br>• Clinique Saint Grégoire – Rennes<br>• CHU Carémeau – Nîmes<br>• Institut Paoli Calmette - Marseille
**BACKGROUND AND RATIONALE**	Breast cancer is the most frequent cancer in women in France [1]. Surgery is, to date, the key treatment for this disease [2]. During care in the operating room, patient arrival is an anxiety-inducing moment, because breast cancer often affects young women, with light or non-existent medical history, taken in a "hostile" environment: noisy, cold, austere, with unknown care pathway … before a surgery for a cancer which is often psychologically-difficult to cope with.<br><br>For all these reasons, premedications using anxiolytiques (benzodiazepin-like) are usually given to decrease patients' anxiety. However, alternative non-pharmacological techniques have shown efficacy in decreasing perioperative pain and anxiety. Among these techniques, sophrology, reinsurance, respiratory techniques and medical hypnosis are among the preferred techniques.<br><br>Regarding hypnosis techniques, many studies have shown that perioperative hypnosis could decrease anesthetic drugs consumption, postoperative pain, length of stay in postoperative care unit (PACU) and numerous postoperative adverse events: nausea, vomiting (PONV), pain, etc…<br><br>Among the different hypnosis techniques proposed to patients in perioperative setting, medical conversational hypnosis consists in supporting the patient as soon as her admission in the operating room to general anesthetic induction. This technique aims at suggesting an agreeable and non-anxietic environment. In a preliminary study in 20 patients [5], which was then confirmed with a randomized trial in 200 patients [11], the authors found that conversational hypnosis, performed by a clinician psychologist 15 minutes before arrival of the patients in the operating room, was associated with a decrease of the postoperative adverse events, mean (CI 95%): PONV (18.9: 12-24), postoperative pain (25: 17-33), asthenia (24: 16-32), discomfort (20: 12-28), emotional charge (24: 18-31).<br><br>However, to date, and to our knowledge, no randomized trial has shown interest and benefit of a conversational hypnosis performed by an anesthesiologist during anesthetic induction.<br><br>We propose in the context of the HYPNOSEIN study to compare perioperative conversational medical hypnosis (hypnosis group) performed by a trained anesthesiologist to a control group with no hypnosis session.

<table>
<tr>
<td>ELIGIBILITY CRITERIA</td>
<td>
<u>Inclusion criteria</u>

- Female > 18 years
- Patient with ASA* score 1 ,2, 3
- Minor Unilateral breast surgery indication (cancer tumorectomy, , tumorectomy with limited axillary node dissection,
- Day case surgery (ambulatory surgery – living Day0-Day1)
- General anesthesia required
- Written informed consent
- French medical benefit

<u>Exclusion criteria</u>

- Age < 18 years
- Patient with ASA score > 4;
- Body mass index < 15 or 45kg/T²;
- Major Surgery indication : mastectomy, bilateral surgery, full axillary dissection, major breast reconstruction, lumpectomy
- Patient refusing hypnosis
- Patient with previous surgery with hypnosis
- Psychic or mental Disorders
- Chronic pain
- Opiate therapeutic > 3 months
- Not ability to speak and read French language
- Deaf and dumb patient
- Under guardianship patient or guardianship

*see appendix
</td>
</tr>
<tr>
<td>TREATMENT MODALITIES</td>
<td>
Patients randomly assigned the day of surgery in one of the two groups:
*Hypnosis group: the conversational hypnosis (10-15 min) is standardized and performed just before intravenous general anesthesia induction in the operative room.
*Control group: no special preparation before intravenous general anesthesia induction in the operative room.

For the groups, preoperative preparation and postoperative care, including analgesia, are similar and standard

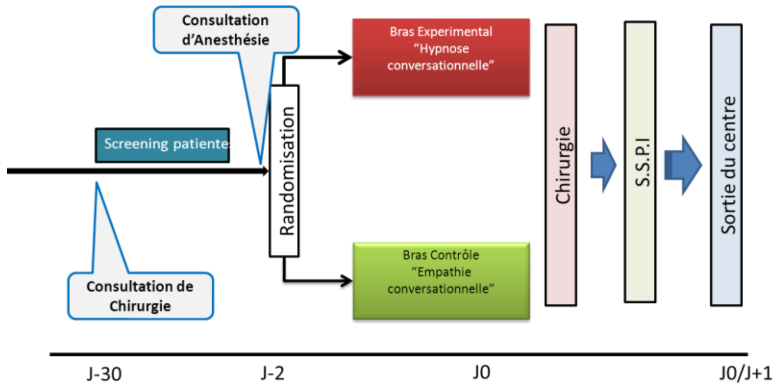

</td>
</tr>
<tr>
<td>OBJECTIVE</td>
<td>Evaluate the impact of Added conversational hypnosis on reduced general anesthesia side effects (pain intensity) for day case breast surgery.</td>
</tr>
</table>

**PRIMARY ENDPOINT**	Reduction of the pain adverse postoperative outcome :<br>Using Visual analogic scale (VAS > 3/10)
**SECONDARY ENDPOINT**	VAS evaluation of :<br>- Post-surgery nausea and vomiting<br>- Fatigue (> 3/10)<br>- Discomfort (> 3/10)<br>- Emotional upset (3/10)<br>Concomitant medication:<br>- Used and dosage of antiemetic's<br>- Analgesic consumption<br>- Failed day case surgery<br>- Clinical times : operating room, post care unit
**STATISTICAL CONSIDERATION RANDOMIZATION**	The proposed study is an interventional multicenter randomized phase III controlled trial. Patients will be randomly allocated (1:1) to receive either the "conversational hypnosis" versus standard conversation. Randomization will be stratified according to Center,<br>Sample size calculation is based on the difference of at least 2 units between the 2 groups in term of Pain severity index (EVA). **150 patients (75 patients /arm**) were planned to be included.<br><br>**Planned number of subjects**<br>The necessary number of subjects was calculated according to the primary endpoint: pain severity measured with a 0 to 10 visual analog scale. The sample size calculation was based on a difference of at least 2 units on the VAS between the two groups in terms of pain severity. To detect such a difference, with an σ =3.5, a bilateral risk α=5% and a power of 90% (β=0.10), 66 patients per group are required. Considering 10% of non-evaluable patients, **a total of 150 patients, 75 per group, are planned.**<br>Calculations were made using the « Sample Size Tables for Clinical Studies Program » (Machin D, Campbell, Beng Tan S, Huey Tan S. Sample size table for clinical studies. Wiley-Blackwell. Comparing two independent groups for continuous data p 47 - Equation 5.2)<br><br>**Statistical analyses**<br>A descriptive analysis per group will be performed. The analyses will be performed on an intention-to-treat basis. Data will be described by treatment group. Continuous variables will be described using means with standard deviations, medians with interquartiles (IQ) according to their distribution. For categorical variables, frequencies and percentages will be computed.<br>It will be checked that the baseline characteristics are well-balanced between the two groups, and that they are thus comparable.

	Efficacy of conversational hypnosis will be assessed comparing the pain severity score of the two groups (bilateral t-test, means and 95% confidence intervals).<br>A similar analysis will be performed using the Student's t-test or the Kruskal-Wallis test to compare the postoperative side-effects measured with a visual analogic scale (discomfort, fatigue, emotional upset) and for all quantitative variables among the secondary endpoints.<br>Standardized values (Δ/σ) will also be presented for each side-effect measured using a VAS.<br>The qualitative secondary endpoints will be compared between the two arms using a Chi-2 test or a Fisher exact test.<br>The analyses will be performed using the Stata v13 software after approval of the statistical analysis plan.

2. ASSESMENT CRITERIA

2.1 PRIMARY ENDPOINT

The objective of the study is to evaluate the impact of added conversational hypnosis performed by a medical anesthesiologist just before anesthetic induction on reduced general anesthesia side effects (pain intensity) for daycase breast surgery.

The primary endpoint will be the pain level assessed after surgery using a Visual analogic scale (VAS > 3/10). The pain score will be assessed at 3 times: Day 0 or 1, Day 7 and D30.

The most relevant evaluation will be the value at Day 0/1.

2.2 SECONDARY ENDPOINTS

The secondary objectives are as follows:

To evaluate the impact of conversational hypnosis on:

- Main postoperative adverse effects:
  - Nausea and vomiting
  - Comfort and well-being
  - Fatigue
  - Anxiety.

- Medical care and medical intake:
  - Analgesic drug consumption including morphinic drugs
  - Antiemetics use
  - Anxiolytics use (such as Benzodiazépine)

- Length of stay in postoperative care unit (PACU)

The secondary endpoints linked to the secondary objectives are as follows:

VAS evaluation of:

- Post-surgery nausea and vomiting (occurrence and score)
- Fatigue (> 3/10)
- Comfort (<7/10)
- Emotional upset (<7/10)

Concomitant medication:

- Used and dosage of antiemetic's
- Analgesic consumption
- Failed day case surgery
- Clinical times : operating room, post care unit
- Classic hospitalization (vs planned daycase surgery)

3. PLANNED ANALYSES

Tableau 1 : Listing of planned analyses

Analysis	Objective	Criteria	Forecast	
Final	Efficacy	Pain EVA	N=150<br>(75 by arms)	January<br>2017

4. DEFINITION OF POPULATIONS

<u>Intent-to-Treat Population (ITT):</u> all included patients, whether treated or not, eligible or not.

<u>Eligible population</u>: all patients without violation of major inclusion or non-inclusion criteria.

<u>Per-protocol population (PP):</u> all eligible patients treated and evaluated.

All statistical analyzes will be performed on the ITT population.
The efficacy analysis will be performed on the PP population.

5. MATERIAL AND METHODS

5.1 DEFINITION AND CONVENTIONS

5.1.1 Conventions

Time to events will be calculated from the date of inclusion.

For any calculation of time between two dates, the following convention will be applied: **[later date] – [earlier date].**

For any calculation of duration between two dates, the following convention will be applied: **[later date] – [earlier date] + 1 jour.**

To convert a number of days to year or month, the following convention will be applied:

1 year = 365.25 days;
1 month = 30.4375 days.

5.1.2 Missing data

Unless otherwise stated, missing values will not be imputed.

If the day of a date is missing, it will be replaced by 15.

5.2 MATERIAL

5.2.1 Subjects disposition

The following will be summarized:
- Rate of inclusion
- Subjects disposition: ITT population, eligible population, PP population
- CONSORT flowchart

The datalistings will be edited and appended in the final report.

5.2.2 Stratification factors

The stratification factor "Center", collected at the randomization will be described..

5.2.3 Baseline characteristics

The initial characteristics (Baseline evaluation) will be described by arms and globally. It will be evaluated between D-30 and D-2.

It corresponds to the demographics (age, education level, socio-economic class), vital signs (height, weight, BMI, blood pressure and pulse), patient characteristics (manual preference, APFEL score (x / 6), Score ASA (x / 4), APAÏS), presence of medical and surgical history → description of chronic pain + depression, presence of surgery with general anesthesia and number of interventions, presence of medical treatments in progress, analogical evaluations (pain breast, general pain, felt pain, nausea / vomiting, fatigue, comfort / well-being, anxiety).

Calculated (and categorized) variables are presented in the following table:

Tableau 2 : Derived variables for baseline characteristics

Variable	Formula
Age	int((date de randomisation – date de naissance) / 365.25)
Age en catégories	Terciles sur la population totale
Indice de masse corporelle (IMC)	IMC= poids/ (taille en m)²<br>4 catégories :<br>maigres <18,5 / normaux<25 / surpoids<30 / obèses≥30
Surface corporelle (SC)	Formule de Dubois and Dubois :<br>SC = (poids en kg) $^{0.425}$ * (taille en m) $^{0.725}$ * 0.20247
Douleur (en catégories)	Code 1 si Douleur (< 3 /10) légère<br>Code 2 si Douleur (≥ 3 /10 & < 7 /10) modéré<br>Code 3 si Douleur (≥ 7 /10) sévère
Nausée/Vomissement (en catégories)	Code 1 si Nausées (< 3 /10) légères<br>Code 2 si Nausées (≥ 3 /10 & < 7 /10) modérées<br>Code 3 si Nausées (≥ 7 /10) sévères
Fatigue (en catégories)	Code 1 si Fatigue (< 3 /10) légère<br>Code 2 si Fatigue (≥ 3 /10 & < 7 /10) modérée<br>Code 3 si Fatigue (≥ 7 /10) sévère
Confort (en catégories)	Code 1 si Confort (< 7/10)<br>Code 2 si Confort (≥ 7/10)
Anxiété (en catégories)	Code 1 si Anxiété (< 3 /10) légère<br>Code 2 si Anxiété (≥ 3 /10 & < 7 /10) modérée<br>Code 3 si Anxiété (≥ 7 /10) sévère
Délai entre Evaluation inclusion et date de chirurgie (J0)	int(date de chirurgie – date de visite évaluation inclusion)

5.2.4 Treatment administration

Treatment administration includes: Hypnosis, Surgery and Anesthesia and will be described by patient.

Hypnosis:

The following items reported will be described only for patients in the hypnosis arm.

- Preoperative description of the mammary lesions (yes, no), if yes, time (D-1, D0).
- Hypnosis technique performed
- If no, reason (listing).
- If yes, duration
- Reasons if duration >15 min
- Listing of elements describing adhesion or non-adhesion to hypnosis
- Adhesion and non-adhesion to hypnosis coded between 1 to 3
- Do you think you have received hypnosis before anesthesia? (Yes, No)

Surgery:

The following items will be described for each arm and for the overall population.

- Surgery type
- Operative time
- Operated side
- Sentinel node associated (yes, no)
- Positive sentinel nodes (yes, no). If yes, axillary node dissection
- Surgery planned as daycase surgery (yes, no)

Anesthesia:

The following items will be described for each arm and for the overall population.

- Vitals signs (anesthesia) (blood pressure, pulse, SPO2)
- Anesthesia duration
- Anesthetics administered and received
- Incubation type
- Nausea and vomiting prophylaxy
- Analgesic
- Vital signs 5 minutes after induction (blood pressure, pulse, SPO2, temperature)
- Should you have another anesthesia, would you choose the same technique? (Yes, no)

Post-interventional surveillance

D0/D1

- Planned duration of stay in PACU
- Real duration of stay in PACU
- Additional treatments and doses received
- Adverse events
- ALDRETE score (<12, ≥12)
- Patients' dream during anesthesia
- Antalgic medication, systematically during 48h (Yes, no) + compliance at D1
- Effective discharge at D0/D1
- Patient's satisfaction related to anesthesia care (0 - 10)

<u>D7</u>

- Should you have another anesthesia, would you choose the same technique? (Yes, no)

<u>J30</u>

- Return to work
- Duration of professionnal inactivity

Tableau 3 : Treatment calculated variables

**Variable**	**Formula**
**By patient**	
Operative time (minutes)	Dressing time
Anesthesia duration (minutes)	Extubation time – Induction time
Intensity of adhesion to hypnosis	Coded according to the number of elements indicating adhesion to hypnosis (1, 2, 3)
Intensity of non-adhesion to hypnosis	Coded according to the number of elements indicating non-adhesion to hypnosis (1, 2, 3)
Theoric duration of stay in PACU (min)	Time of discharge decided by the anesthesiologist – Arrival time in PACU
Real duration of stay in PACU (min)	Discharge time (from the OR) – arrival time in PACU
Duration of professionnal inactivity (days)	Date of return to work - Date of surgery

5.2.5 Toxicity evaluation

5.2.5.1 Adverse events

All adverse events of grade ≥ 3 will be described by toxicity type. Severity of the Aes will be graded according to the NCI-CTCAE scale (version 4.0).

AEs will be described by patient.
The number of patients who underwent at least one AE will be described.
Every AE will be described according to the following groups :

- 3 grades (3,4,5),

If an AE is reported more than once during treatment, the higher grade will be reported for that given patient.

Datalistings will be edited and reported in appendix in the final report.

5.2.6 Efficacy evaluation

5.2.6.1 Primary endpoint

The primary criterion defined in subsection 2.1 (critère quantitatif) will be analyzed according to the methods described in paragraph 5.3.1.1.

Means and medians for the pain VAS scores at all times will be compared between the two arms.

5.2.6.2 Secondary endpoints

Secondary quantitative endpoints defined in subsection 2.2 will be analyzed according to the methods descirbed in paragraph 5.3.1.1.

Secondary qualitative endpoints defined in subsection 2.2 will be analyzed according to the methods descirbed in paragraph 5.3.1.2.

5.2.7 Concomitant treatments

The number of patients with at least one concomitant treatment will be described.

The datalistings will be edited and appended in the final report.

5.3 STATISTICAL METHODS

The analyzes will be carried out by treatment arm and globally (phase III).

All statistical tests are bilateral and the significance threshold is set at 5% (ie $p < 0.05$).
Statistical analyses will be carried out using the STATA v13.0 software and a statistical report will be provided.

5.3.1 Descriptive statistics

5.3.1.1 Continuous variables

The continuous variables will be described by the number of observations (N), the median, the minimum, the maximum, the mean and the standard error. Student t test and Kruskal-Wallis test will be used to compare the quantitative variables distribution.

Pain VAS scores between Day 0 (or Day 1) and D7 will be compared in each group using a Wilcoxon test (case-matched samples)

Optional: longitudinal analyses with linear mixt models will be performed for the pain VAS scores.

5.3.1.2 Categorical variables

The categorical variables will be described by the number of observations (N) and the frequency (%) of each modality. The missing categories will be counted.

Percentages will be calculated in relation to the total population excluding missing data.

The Chi-2 test will be used to compare proportions (or Fisher's exact test if the expected frequencies are less than 5).

5.3.2 Survival data

Not applicable.

6. APPENDIX

6.1 APPENDIX 1: LIST OF TABLES AND DATA LISTINGS

6.1.1 Baseline

N°	Légende	Paramètres décrits	Forme*
1.0	Participating centers	Centers	T
2.0	Analysis populations	Included, ITT, treated, Evaluable for tolerance	T+DL
3.0	Deviations	Reasons, concerned criteria	T+DL
4.0	Patient characteristics	Age, study level, socio-economic level, size, weight, BMI, blood pressure, pulse, APFEL score, ASA score, APAÏS score.	T
5.0	Medical and surgical history at baseline	Medical history: chronic pain and depression	DL
6.0	Analogic evaluation at baseline (quantitative and qualitative)	Breast pain, general pain, nausea/vomiting, fatigue, comfort/well-being, anxiety.	T

6.1.2 Treatment

N°	Légende	Paramètres décrits	Forme*
7.0	Hypnosis	All data relative to hypnosis	T
8.0	Surgery	All data relative to surgery	T
9.0	Anesthesia	All data relative to anesthesia	T
10.0	PACU surveillance	PACU surveillance at D0/D1, D7 and D30	T

6.1.3 Toxicity evaluation

N°	Légende	Paramètres décrits	Forme*
11.0	Adverse events	General listing global of all grade ≥ 3 adverse events (AEs) reported.	T+DL

6.1.4 Efficacy

N°	Légende	Paramètres décrits	Forme*
12.0	Efficacy: primary endpoint	Breast pain VAS score	T+DL
13.0	Efficacy :secondary endpoints	Analysis of all secondary endpoints as defined in the protocole	T+DL

6.1.5 End of study

N°	Légende	Paramètres décrits	Forme*
	Reasons for end of study	Reasons for end of study, death (cause), description	T+DL

T= Table

DL= Data listing
